# Direct Synthesis of Formamide from CO_2_ and H_2_O with Nickel−Iron Nitride Heterostructures under Mild Hydrothermal Conditions

**DOI:** 10.1021/jacs.3c05412

**Published:** 2023-08-29

**Authors:** Tuğçe Beyazay, William F. Martin, Harun Tüysüz

**Affiliations:** Max-Planck-Institut fur Kohlenforschung, 45470 Mulheim an der Ruhr, Germany; Institute of Molecular Evolution, University of Dusseldorf, 40225 Dusseldorf, Germany; Max-Planck-Institut fur Kohlenforschung, 45470 Mulheim an der Ruhr, Germany

## Abstract

Formamide can serve as a key building block for the synthesis of organic molecules relevant to premetabolic processes. Natural pathways for its synthesis from CO_2_ under early earth conditions are lacking. Here, we report the thermocatalytic conversion of CO_2_ and H_2_O to formate and formamide over Ni−Fe nitride heterostructures in the absence of synthetic H_2_ and N_2_ under mild hydrothermal conditions. While water molecules act as both a solvent and hydrogen source, metal nitrides serve as nitrogen sources to produce formamide in the temperature range of 25−100 °C under 5−50 bar. Longer reaction times promote the C−C bond coupling and formation of acetate and acetamide as additional products. Besides liquid products, methane and ethane are also produced as gas-phase products. Postreaction characterization of Ni−Fe nitride particles reveals structural alteration and provides insights into the potential reaction mechanism. The findings indicate that gaseous CO_2_ can serve as a carbon source for the formation of C−N bonds in formamide and acetamide over the Ni−Fe nitride heterostructure under simulated hydrothermal vent conditions.

**Figure:**
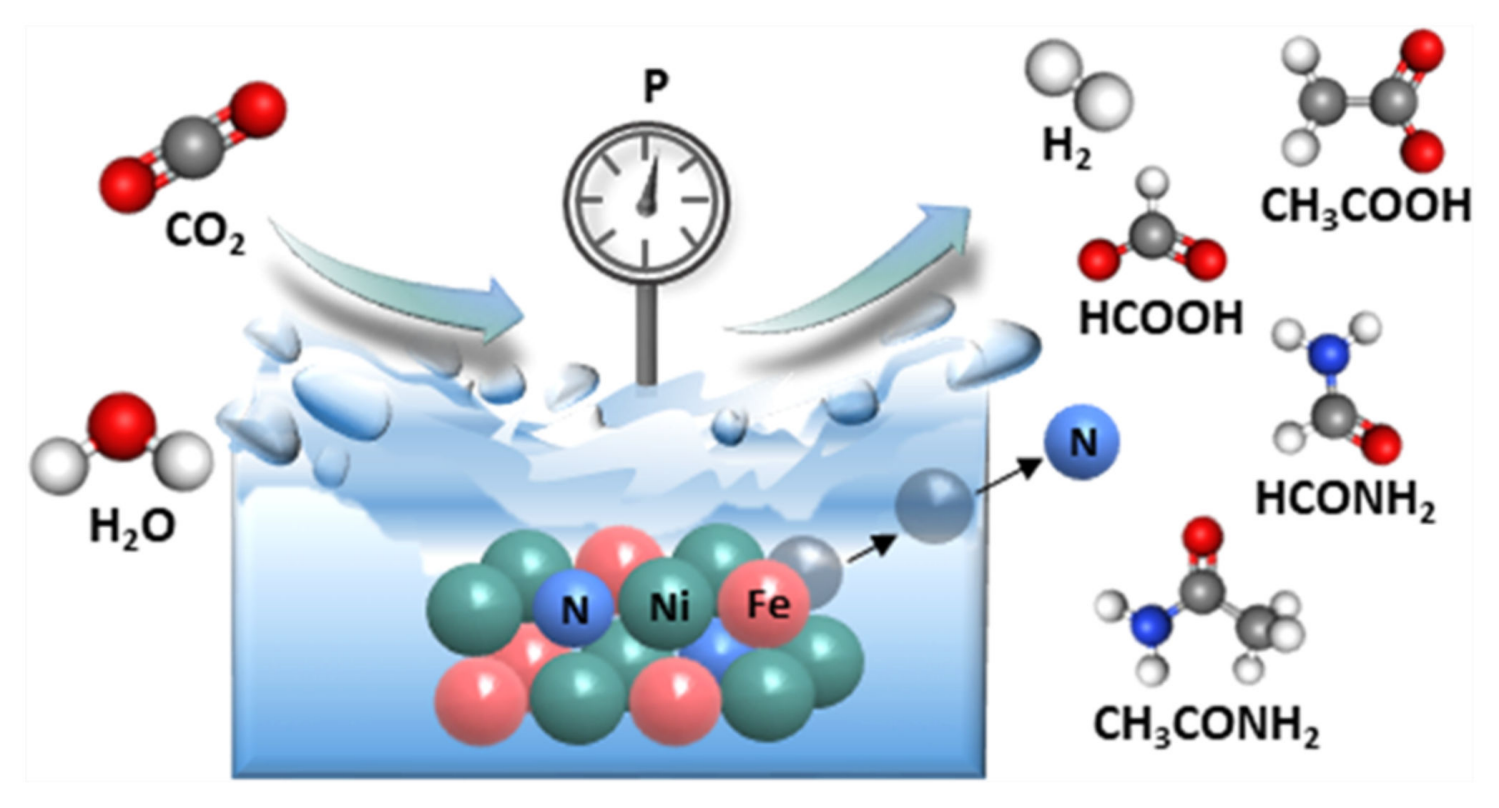

## Introduction

Amides are an important class of compounds in biological and chemical sciences.^1^ They have been used in the manufacture of pharmaceuticals and agrochemicals, and they are also the basis of some versatile synthetic polymers.^2^ C–N coupling reaction that produces nitrogenous compounds including amides is one of the most fundamental reactions in the chemical industry.^3–5^ In life, amide functional groups are ubiquitous moieties in amino acids and the peptide bond.^6^ Formamide, the simplest amide, consists of the most common elements (C, H, N, and O) in the universe and is widely used for the synthesis of prebiotic molecules.^7,8^ Formamide can be considered as a multifunctional tool for prebiotic chemistry since its condensation and degradation products generate—in the presence of minerals and metal oxides—biologically relevant molecules including amino acids, cofactors, nucleobases, and carboxylic acids.^7,9,10^ Formamide produces the nucleosides adenine, purine, hypoxanthine, cytosine, thymine, and uracil.^11^ In 2008, Saladino et al. reported that the interaction of formamide with the hydrothermal vent mineral pyrite (FeS_2_) yields purine and adenine,^12^ which are basic components of nucleic acids.^8,13^ Formamide was also reported to serve as a solvent for the phosphorylation of nucleosides to nucleotides.^14^ A recent study by Green et al. demonstrated the formation and the further conversion of aminonitriles in formamide.^15^ Furthermore, degradation products of formamide, formic acid, formaldehyde, HCN, ammonia, and CO_x_ serve as substrates for the synthesis of other intermediates in prebiotic chemistry including sugars.^16,17^ Formamide is not only a parent molecule but also an intermediate in a series of reactions from very reactive small radicals to biologically significant molecules^9^ as implicated in Miller’s classical 1953 experiment.^18^ Computational studies suggested that formamide is a key intermediate in the Miller synthesis of glycine.^19^ Although a reducing gas mixture consisting of NH_3_, CH_4_, and H_2_ was used in Miller’s experiment, a more oxidizing early atmosphere composed of N_2_, CO_2_, and H_2_O is predicted by many geoscientists.^20^ Overall, formamide is a versatile compound in prebiotic chemistry that can generate a range of monomers, from amino acids to nucleic acids.

The synthesis of formamide under early earth conditions is of interest. Its formation has been studied starting from CO and NH_3_ with UV light, from the conversion of aqueous acetonitrile by *γ*-irradiation, electrochemical synthesis from formic acid, and the electrosynthesis from methanol and ammonia.^21–24^ It can also be formed from formic acid and ammonia.^9^ Proposed reactions for the formation of formamide generally entail the presence of minerals, temperatures higher than 100 °C, and relatively high pH values, which are compatible with hydrothermal vent conditions.^9^ Additionally, it has been shown that the accumulation of formamide in hydrothermal vents via thermophoresis is possible.^25^ Its constituents are formate and ammonia. The former is common in hydrothermal vents, and the latter can be generated under hydrothermal vent conditions.^26,27^ The fixation of dinitrogen to ammonia in the presence of H_2_S and FeS under mild conditions (under atmospheric pressure at 70−80 °C), which are close to the biological conditions, has been experimentally simulated and shown to be feasible.^28^ In addition, recent laboratory-scale serpentinization reactions of peridotite, water, and N_2_ generated up to 2 *μ*mol of NH_3_ per gram of peridotite after 30 days at 250 °C, with synthesis rates accelerated up to 10-fold with the addition of CO_2_.^29^ The formation of formate in hydrothermal vents is driven by the serpentinization process, which yields H_2_ for the reduction of CO_2_ and its dissolved forms.^30^ The oxidized carbon species are presumed to be the ultimate source of carbon for the abiotic synthesis of organic molecules.^31,32^ Several studies report the formation of formate from CO_2_ with hydrothermal minerals under mild hydrothermal vent conditions.^33–36^ The formation of formate and acetate occurs via the gas-phase H_2_-dependent CO_2_ reduction over silica-supported Co nanoparticles,^37^ and Ni–Fe nano-particles are able to reduce CO_2_ to the free intermediates of the acetyl-CoA pathway of CO_2_ fixation—formate, acetate, and pyruvate—under mild hydrothermal conditions in the absence of the synthetic H_2_.^38^ The conditions of serpentinizing systems are reducing enough to have stable forms of Ni–Fe alloys and their native metal forms.^39,40^ Awaruite (Ni_3_Fe) is known to exist in H_2_-rich serpentinizing systems.^41,42^ Furthermore, a recent study by Peters et al. reported that nickel−iron-containing meteoritic catalyst (Campo del Cielo) yields methanol, ethanol, acetaldehyde, and alkanes from the hydrogenation of CO_2_ under hydrothermal conditions.^43^

While oxidized carbon species have been suggested to be the source of primordial carbon fixation pathways, there have been several proposals for the abiotic formation of reduced nitrogen species.^27,44,45^ One of the proposed sources of nitrogen is the release of N_2_ or NH_3_ from rocks and minerals, presumably from ammonium silicates or metal nitrides.^46^ In addition, the existence of the nitride mineral, siderazot, as a terrestrial mineral has been reported.^47^ Since the direct incorporation of N_2_ gas into the CO_2_ fixation system is very challenging due to the strong triple bond of N_2_ (with the bond dissociation energy of 945 kJ/mol),^48^ modified metal catalysts with chemisorbed nitrogen would be a better starting point to investigate the possible formation of nitrogenous compounds of prebiotic significance. Furthermore, early atmosphere has been assumed to contain more oxidizing species including CO_2_ and H_2_O rather than reduced species.^20^ Thus, the formation of formamide from CO_2_ and H_2_O with metal nitrides under hydrothermal vent conditions is very interesting.

Here, we report the preparation of Ni−Fe nitride heterostructures via NH_3_ treatment of metallic nanoparticles and their implementation as a thermal catalyst for CO_2_ fixation under mild hydrothermal conditions. The mixed phase of the Ni_3_FeN/Ni_3_Fe structure yielded formate and formamide in water in the absence of synthetic H_2_ and N_2_. Effects of reaction parameters including reaction temperature, initial pH, CO_2_ pressure, and the reaction time on the product formation are systematically studied. Postreaction analysis of Ni−Fe nitride particles sheds light on possible reaction mechanisms and the nitrogen source for the amide formation.

## Results and Discussion

We utilized metal−metal nitride heterostructure as a catalyst and substrate to convert CO_2_ and water to amides in a single step. Direct synthesis of metal nitrides from N_2_ gas is challenging due to the stability of molecular N_2_.^49^ On the other hand, nitridation of transition metals and their oxides under a gas flow of ammonia is a well-established process.^50^ Properties of the resulting metal nitrides depend on several factors including synthesis temperature, heating rate, and flow rates. To find optimal annealing temperatures, in situ X-ray diffraction (XRD) patterns of the Ni_3_Fe sample were collected under an ammonia flow in the temperature range of 30−400 °C. As seen in Figure S1, Ni_3_FeN formation began at a temperature of about 300 °C. Higher treatment temperatures as 400 °C can result in the catalytic decomposition of NH_3_ to N_2_ and H_2_, which is an undesirable byproduct due to its reducing effect.^51^

After observing the optimum temperature range of 300−380 °C for ammonia treatment by in situ XRD, the treatment conditions were systematically varied to adjust the composition of the catalyst. First, a bimetallic Ni_3_Fe sample was nitrided under an ammonia atmosphere in a quartz tube at 300 and 350 °C for 1 and 2 h. As shown in Figure S2, annealing at 300−350 °C for 1 or 2 h results in a heterostructure that consists of crystalline Ni_3_FeN and Ni_3_Fe phases while Ni_3_Fe remained as the major phase. Reflection indices of (111), (200), and (220) planes could be assigned to the Ni_3_FeN (PDF: 00-050-01434) crystalline phase. The incorporation of more electronegative N atoms into parent metal structures increases the atomic distance between metal atoms in the crystal lattice. Therefore, reflections of Ni_3_FeN appear at lower 2*θ* values compared to that of the Ni_3_Fe alloy structure. The reflection ratios of Ni_3_FeN to Ni_3_Fe were not altered significantly by changing the annealing temperature from 300 to 350 °C or increasing the treatment time from 1 to 2 h.

After confirming the formation of the Ni_3_FeN/Ni_3_Fe heterostructure by XRD, textural parameters and elemental compositions of two selected samples (Ni_3_FeN/Ni_3_Fe-300-2h and Ni_3_FeN/Ni_3_Fe-350-2h) were further investigated by N_2_-sorption and scanning electron microscopy−energy-dispersive X-ray (SEM−EDX) spectroscopy, respectively. N_2_-sorption isotherms show hysteresis, which is related to the condensation of nitrogen within the interparticle porosity. The hysteresis loop in N_2_-sorption isotherms was maintained after the mild nitridation treatment at 300 and 350 °C; the Ni_3_Fe morphology was not altered noticeably. The Brunauer−Emmett−Teller (BET) surface areas were found to be 28 and 27 m^2^/g for Ni_3_FeN/Ni_3_Fe-300-2h and Ni_3_FeN/Ni_3_Fe-350-2h, respectively (Figure S3). Figure S4 displays the large-area (250 *μ*m resolution) scanning electron microscopy−energy-dispersive X-ray (SEM−EDX) spectroscopy elemental mapping of these selected heterostructures with the distributions of Ni, Fe, and N atoms. The Ni_3_FeN/Ni_3_Fe-300-2h sample has a slightly higher N content (7.4 atom %) than Ni_3_FeN/Ni_3_Fe-350-2 h (6.8 atom %). SEM−EDX mapping performed at a 100 nm range for the selected sample of Ni_3_FeN/Ni_3_Fe-350-2h indicated a homogeneous distribution of Ni, Fe, and N atoms (Figure S5).

**Figure 1:**
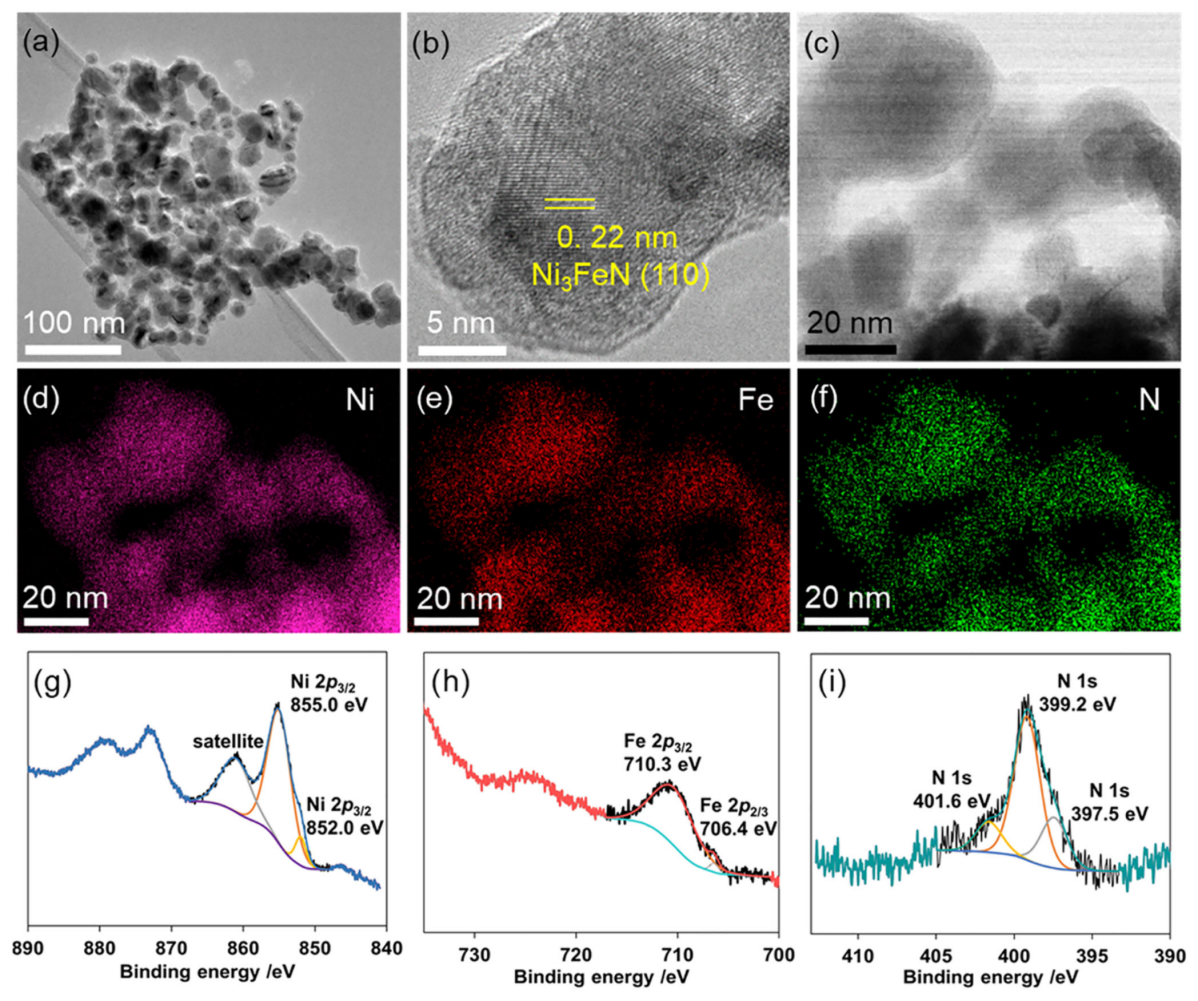
TEM (a), HR-TEM (b), STEM (c), corresponding STEM−EDX elemental mapping (d−f), Ni 2p (g), Fe 2p (h), and N 1s (i) spectra of Ni_3_FeN/Ni_3_Fe prepared at 350 °C for 2 h.

The morphology of the selected Ni_3_FeN/Ni_3_Fe-350-2h sample was further analyzed by transmission electron microscopy (TEM) where nanoparticles in the range of 15−30 nm could be imaged (Figures 1a and S6). After ammonia treatment, they maintained their initial morphology and shape. As seen in Figure 1b, high-resolution TEM (HR-TEM) imaging further supports the formation of crystalline Ni_3_FeN with an interplanar spacing of 0.22 nm, which corresponds to the (111) plane of Ni_3_FeN. Moreover, scanning transmission electron microscopy (STEM)−EDX mapping demonstrated the homogeneous distribution of N, Ni, and Fe atoms in the selected field (Figure 1c−f). More direct evidence of the homogeneous distribution of Ni, Fe, and N elements was obtained by STEM−EDX line scanning analysis along a linear path passing through central and peripheral parts of two arbitrary representative nanoparticles for the Ni_3_FeN/Ni_3_Fe-350-2h sample (Figure S7). Line scanning profiles of the composite particles show that Ni, Fe, and N signals are located homogeneously across the particles. The surface chemistry and composition of the selected Ni_3_FeN/Ni_3_Fe-350-2h heterostructure were probed by X-ray photoelectron spectroscopy (XPS). As shown in Figure 1g, the Ni 2p spectrum can be convoluted into two main features at 852.0 and 855.0 eV, which can be assigned to Ni^0^ and Ni^2+^ species, respectively.^52^ The satellite peak around 860 eV corresponds to the shake-up excitation of the high-spin nickel ions.

Figure 1h displays a high-resolution Fe 2p spectrum, which can be convoluted into two main peaks at 706.4 and 710.3 eV that can be assigned to metallic Fe and Fe^3+^ species, respectively.^53,54^ The reason for the formation of Fe^3+^ species on the metallic Fe surface could be the fast surface oxidation after the nitridation experiment. Furthermore, the N 1s spectrum of Ni_3_FeN/Ni_3_Fe-350-2h is shown in Figure 1i. The N 1s spectrum displays one peak, which can be convoluted into three components at 397.5, 399.2, and 401.6 eV that correspond to the nitrided composition.^53^ While the peak at 397.5 eV matches with the complete transformation to Ni−Fe nitride species,^53^ peaks at 399.2 and 401.6 eV can be assigned to −NH and −NH_2_ surface moieties after the ammonia treatment, respectively.^52,55^ It can be concluded that the surface structure of Ni_3_FeN/Ni_3_Fe-350-2h consists of metal nitride species in addition to metallic Ni and Fe. In addition to bimetallic Ni_3_FeN/Ni_3_Fe heterostructures, mixed phases of monometallic nickel nitride and iron nitride were also prepared via ammonia treatment at 300 and 350 °C, respectively (Figure S8) to reveal differences in catalytic performances. Although heterostructures of monometallic Fe nitrides and bimetallic Ni_3_Fe nitrides were successfully synthesized, this could not be achieved for Ni nitrides since it decomposes at a synthesis temperature of 350 °C due to the lower decomposition temperatures of nickel nitride.^56^ The XRD pattern of Ni samples treated at 300 °C for 2 h (Ni_3_N/Ni-300) shows that Ni is still the main phase and small diffractions were observed at 38.9, 42.1, 44.5, and 58.5° corresponding to (110), (002), (111), and (112) planes of Ni_3_N (PDF: 00-010-0280), respectively. As shown in Figure S8b, Fe_*x*_N/Fe structures were successfully synthesized, Fe_4_N (PDF: 00-006-0627) being the main component in addition to Fe_3_N (00-049-1662).

After the structural analyses, the catalytic performances of the heterostructures were further investigated for CO_2_ and H_2_O conversion to oxygenates and amides by using a pressurized autoclave system, which can operate up to 400 bar and 300 °C. The experimental setup is shown in Figure S9. The analyses of the formed liquid products were done by proton nuclear magnetic resonance (^1^H NMR) and high-performance liquid chromatography (HPLC). Initially, standards of expected nitrogen and carbon fixation compounds were measured by ^1^H NMR spectroscopy and results are displayed in Figures S10 and S11, respectively. Before the thermocatalytic survey, a series of control experiments were conducted to explore the potential catalytic background of the reactor system. First, a reaction was performed to check possible contaminations from the catalyst with water under 25 bar of Ar at 100 °C for 16 h. Another control reaction was performed with CO_2_ and H_2_ gases (25 bar, 3:2 ratio) at 100 °C in the absence of the metal catalyst. No formamide formation was detected, while a very low concentration of formate was found in control experiments (Figure S12). The trace formate might be possible contaminations from the air dissolved in Milli-Q water that was used as a solvent although the system was purged with Ar prior to the experiment.

With potential contamination sources from the reactor system characterized as negligible, thermocatalytic CO_2_ fixation was performed in 2 mL of H_2_O under 25 bar of CO_2_ at 100 °C for 16 h over 0.50 mmol (122 mg) of bimetallic Ni_3_FeN/Ni_3_Fe-350-2h and Ni_3_FeN/Ni_3_Fe-300-2h catalysts. The product analyses through ^1^H NMR revealed the generation of formate and formamide over both of the catalysts after 16 h at 100 °C (Figure S13). Since the reactions were performed in H_2_O in the absence of synthetic H_2_ and N_2_, nitrogen and hydrogen for the formation of formamide can be provided solely by Ni_3_FeN/Ni_3_Fe heterostructures and water, respectively. It is known that formate can be obtained from CO_2_ via autocatalysis mechanisms in water over transition metals.^57^ A similar phenomenon has also been observed, and H_2_O was transformed into formate and acetate over metallic Ni_3_Fe nanoparticles.^38^ This phenomenon and the formation pathway of nitrogen-containing compounds is discussed below in detail. The Ni_3_FeN/Ni_3_Fe-300-2h and Ni_3_FeN/Ni_3_Fe-350-2h show district catalytic differences for CO_2_ fixation. Ni_3_FeN/Ni_3_Fe-350 yielded formate and formamide as main products in the concentrations of 2.06 and 0.77 mM, respectively, under 25 bar and 100 °C (Figure S13c). With the Ni_3_FeN/Ni_3_Fe-300 heterostructure, amounts of formate and formamide decreased significantly to 0.65 and 0.08 mM, respectively. In addition to C_1_ products, acetate and acetamide were obtained over Ni_3_FeN/Ni_3_Fe-300-2h after 16 h. The slightly higher amount of nitrogen in Ni_3_FeN/Ni_3_Fe-300-2h (based on SEM−EDX analyses) might facilitate the C−C coupling toward the formation of C_2_-compounds. It is known that introducing nitrogen atoms to the parent metal structure changes the energies of adsorption and desorption behaviors of reaction intermediates. The electronegative nitrogen atom alters the d-band energy density of the parent transition metal and improves the activity for electron donation reactions.^58^ Previously, it was suggested by Moran and colleagues that acetate is formed via a formate pathway.^59^ Therefore, it is possible that formate was converted to acetate and therefore the low concentration of formate was detected with the Ni_3_FeN/Ni_3_Fe-300-2h sample.

**Figure 2:**
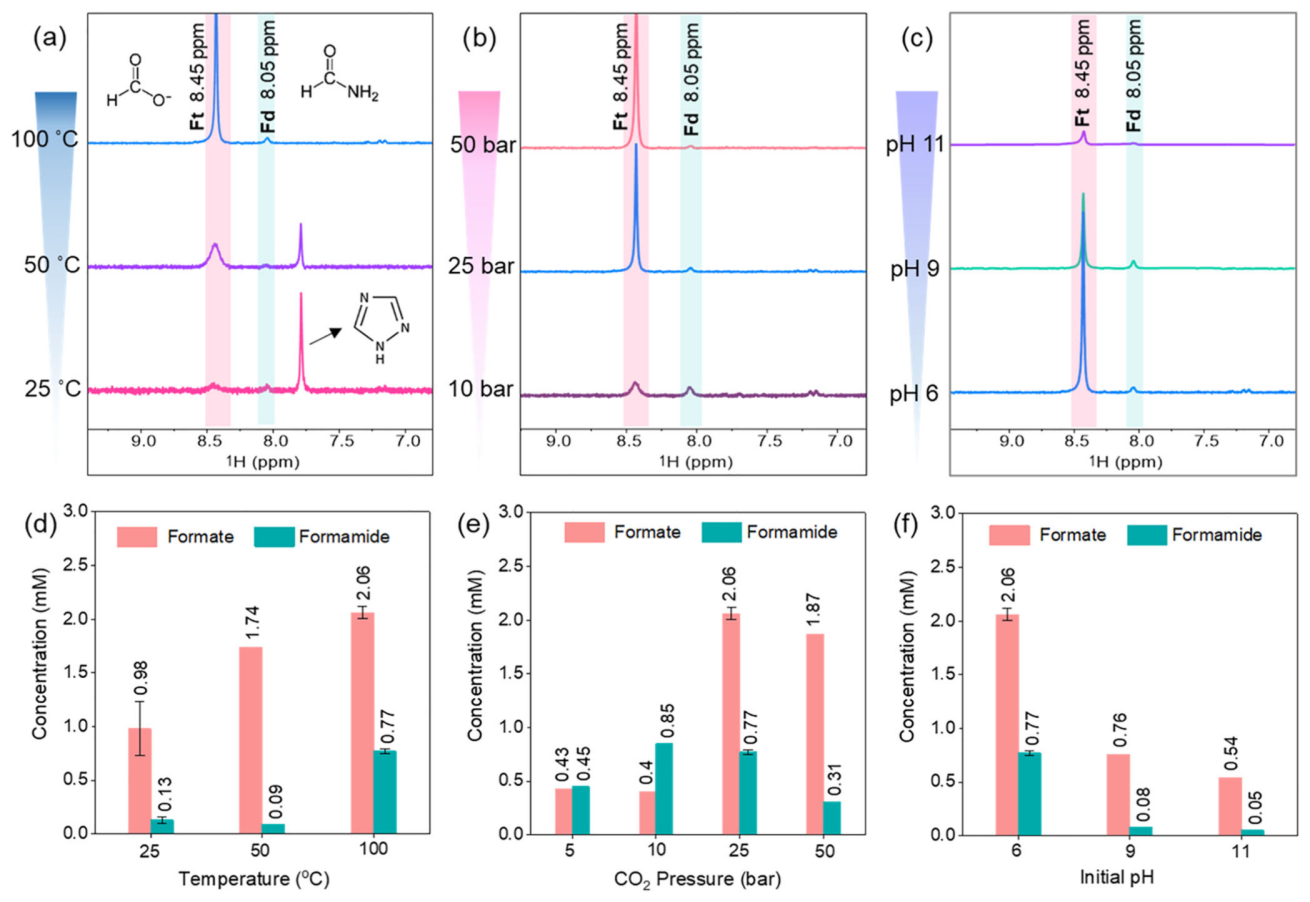
^1^H NMR spectra of the products (with their molecular structures) obtained under 25 bar CO_2_ at different temperatures (a), under different initial CO_2_ pressures at 100 °C (b), and at different initial pH values under 25 bar of CO_2_ at 100 °C (c) over the Ni_3_FeN/Ni_3_Fe-350-2h heterostructure after 16 h in H_2_O. Concentrations of obtained products (calculated from related ^1^H NMR spectra) at different temperatures (d), diverse initial CO_2_ pressures (e), and different reaction pH values (f). Ft: formate, Fd: formamide. Error bars represent the standard deviations of at least two independent reactions.

After testing bimetallic Ni_3_FeN/Ni_3_Fe heterostructures, monometallic Ni_3_N/Ni and Fe_*x*_N/Fe heterostructures were further tested under the same reaction conditions, 25 bar of CO_2_ at 100 °C for 16 h, to see performances of counterparts of the Ni_3_Fe alloy. ^1^H NMR results of the obtained liquid products are shown in Figures S14 and S15. In both cases, formamide was not observed. Fe_*x*_N/Fe yields very low amounts of carbon fixation products that were not detectable by HPLC (Figure S14). However, the formation of acetate and acetamide was promoted over Ni_3_N/Ni particles (Figure S15). While 0.43 mM of formate was obtained over Ni_3_N/Ni particles, which is almost one-fifth of the amount obtained over Ni_3_FeN/Ni_3_Fe particles, concentrations of acetate and acetamide were 0.07 and 0.19 mM, respectively. Overall, formamide formation was detected over only bimetallic heterostructures. The reason for this trend is discussed below in the catalyst alteration part in more detail. After observing that monometallic Ni_3_N/Ni and Fe_*x*_N/Fe heterostructures did not yield a significant amount of formamide, bimetallic Ni_3_FeN/Ni_3_Fe was chosen as a substrate and a catalyst for CO_2_ fixation reactions. Due to the higher formamide selectivity with the Ni_3_FeN/Ni_3_Fe-350-2h heterostructure compared to that of Ni_3_FeN/Ni_3_Fe-300-2h, effects of reaction parameters on the product formation were further investigated by varying the temperature, the initial pH of the solution, and the initial CO_2_ pressure by using Ni_3_FeN/Ni_3_Fe-350-2h. As seen in ^1^H NMR spectra in Figure 2a, both formate and formamide were detected with the Ni_3_FeN/Ni_3_Fe-350-2h catalyst at temperatures of 25, 50, and 100 °C under 25 bar of initial CO_2_ pressure after 16 h. Although the dissolution of CO_2_ increases with decreasing temperatures in water, the obtained formate and formamide amounts were lower at 25 and 50 °C compared to 100 °C (Figure 2d). The reason might be related to the formation of an additional compound peak at 7.79 ppm in ^1^H NMR (Figure 2a) at temperatures of 25 and 50 °C. This new peak at 7.79 ppm was assigned to 1,2,4-triazole, an aromatic nitrogen heterocycle with the formula C_2_N_3_H_3_.^60^ Computational studies of the possible formation of purine from formamide suggested that the ring closure reaction of formamide is thermodynamically favorable in the presence of water.^10^ Therefore, formamide generated from CO_2_ and nickel−iron nitrides can be a building block for the formation of this type of cyclic nitrogen compound at low reaction temperatures. Another way to improve the solubility of CO_2_ in water is by increasing the partial pressure of CO_2_ according to Henry’s law.^61^ We performed a set of reactions under different initial CO_2_ pressures in the range of 5–50 bar at 100 °C with a Ni_3_FeN/Ni_3_Fe-350-2h catalyst. As displayed in ^1^H NMR spectra in Figure 2b, formate and formamide were detected in all pressure values at 100 °C. Increasing the partial pressure of CO_2_ to 25 bar from 5 bar led to the enhancement of the amount of formate and formamide from 0.43 to 2.06 mM and from 0.45 to 0.77 mM, respectively. However, a further increase in the initial CO_2_ pressure to 50 bar results in the decrease of formate and formamide concentrations to 1.87 and 0.31 mM, respectively (Figure 2e). The reason might be associated with the excess amount of CO_2_ that can block/occupy the active center of the Ni_3_FeN/Ni_3_Fe catalyst and causes its sudden deactivation, which is discussed below along with the catalyst alteration. Reaction pH is another key factor that can affect the solubility of CO_2_ and its interaction with the solid catalysts, as well as the product spectrum.^62^ The effect of the initial pH of the reaction solution was investigated at a series of pH values from 6 to 11 (Figure 2c). The increase in the initial pH of the reaction solution resulted in the decrease of formate and formamide amounts from 2.06 to 0.54 mM and from 0.77 to 0.05 mM, respectively (Figure 2f). Notably, the formamide yield was about 10 times higher at pH 6. There could be several reasons for this trend: (i) change of carbonic acid equilibrium in water at different pH values, CO_3_^2−^ ions tend to be formed in alkaline media,^63^ and different CO_2_ forms in the aqueous phase at different pH values have different solubilities,^57^ (ii) potential decomposition of obtained compounds under alkaline conditions, especially hydrolysis of formamide in alkaline aqueous solutions can occur via the nucleophilic attack of an amide bond by hydroxide ion,^64,65^ (iii) catalyst alteration and stability of nitrides might be varied at different pH values.

**Figure 3:**
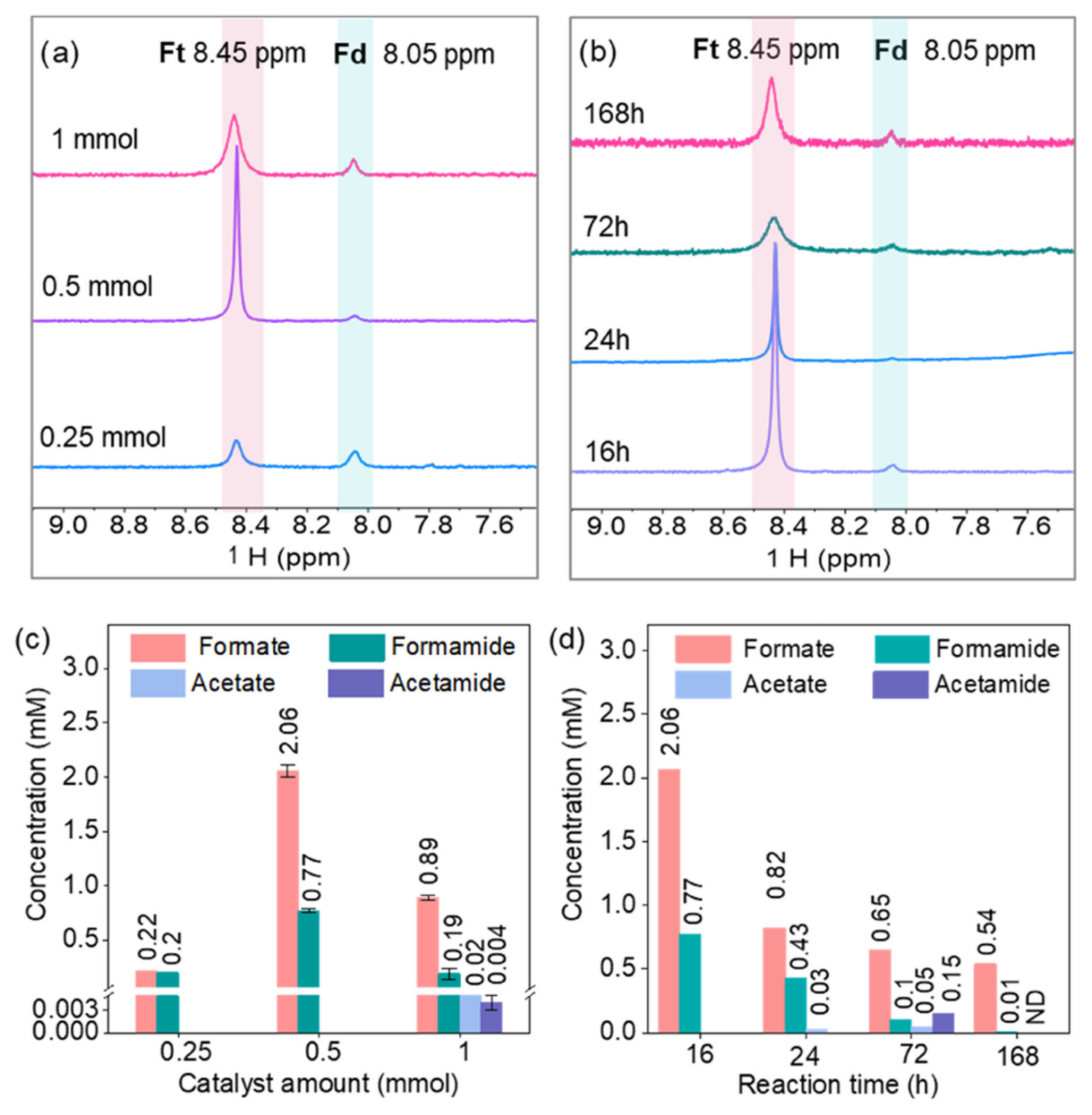
^1^H NMR spectra of the products obtained with different amounts of the catalyst under 25 bar CO_2_ for 16 h (a) and products after different reaction times with 0.5 mmol catalyst 25 bar CO_2_ at 100 °C (b), concentrations of obtained products with different amounts of catalysts, obtained from ^1^H NMR spectra in panel (a) (c), and after different reaction times, obtained from ^1^H NMR spectra in panel (b) (d). Ft: formate, fd: formamide, ND: not detected. Error bars represent the standard deviations of at least two independent reactions.

After observing that the Ni_3_FeN/Ni_3_Fe-350-2h heterostructure yielded the highest amounts of products at the optimized conditions of 25 bar of CO_2_ at pH 6 and 100 °C for 16 h, the impacts of the catalyst amount and the reaction time on product formation were further examined. In addition to the initial amount of 0.5 mmol (122 mg) of Ni_3_FeN/Ni_3_Fe-350-2h nanoparticles, 0.25 mmol (61 mg) and 1 mmol (244 mg) of the solid catalyst were tested under 25 bar of CO_2_ at 100 °C for 16 h. The ^1^H NMR spectra in the range of 7.5–9.5 ppm are provided in Figure 3a, and the whole spectral range is presented in Figure S16. As seen in Figure 3c, decreasing the catalyst amount from 0.5 to 0.25 mmol results in a decline in product concentrations (from 2.06 to 0.22 mM formate and from 0.77 to 0.2 mM formamide). Since H_2_ can be obtained via the interaction of the metal with water due to water dissociation, the increase in metal loading increases the H_2_ amount that shifts the reaction equilibrium toward products according to Le Chatelier’s principle. In addition, formic acid decomposition can occur at high temperatures via the decarboxylation pathway in aqueous solutions, which yield CO_2_ and H_2_ as products.^66^ Reaction equilibrium of formic acid decomposition can shift to the reactant side due to a higher amount of H_2_. However, a further increase in Ni_3_FeN/Ni_3_Fe-350–2h amounts to 1 mmol results in a decrease in formate and formamide concentrations (from 2.06 to 0.89 mM formate and from 0.77 to 0.19 mM formamide). Acetate and acetamide were detected as additional products with concentrations of 0.02 and 0.004 mM, respectively. To gain some insights into reaction intermediates and mechanism, CO_2_ fixation was performed at longer reaction times of up to 7 days under 25 bar of CO_2_ at 100 °C. Figure 3b displays the formate and formamide regions in ^1^H NMR; the whole ^1^H NMR spectra are provided in Figure S17. The quantitative analysis results of the obtained products are plotted in Figure 3d. When the reaction time is increased from 16 to 24 h, formate and formamide concentrations decreased to 0.82 and 0.43 mM, respectively, and acetate is formed as a new product (0.03 mM). This hints that formate is a substrate for the formation of acetate. When the reaction time was extended to 72 h, acetamide formation (0.15 mM) was confirmed. While the amounts of acetate and acetamide were enhanced up until 72 h, a further increase in the reaction time to 168 h led to a significant decrease in all product concentrations, likely due to product decomposition in aqueous media at high temperature and pressure. Formic acid and formamide are known to decompose to lower-molecular-weight compounds in water.^16,66^ In addition to liquid products, gas products of the reaction were also analyzed by gas chromatography (GC). Gas product analysis after a reaction time of 16 h reveals that methane and ethane were produced with 25 bar CO_2_ at 100 °C using the Ni_3_FeN/Ni_3_Fe-350h-2h heterostructure (Figure S18a). There was no detectable additional gas product as the reaction time increased to 72 h (Figure S18b).

In previous studies with NiFe catalysts, it was shown that the addition of H_2_ promotes formate formation from CO_2_.^33,67^ A control reaction was conducted with the addition of 10 bar of H_2_ gas to study the role of H_2_ versus water as a reductant. The catalytic reaction was performed with 0.5 mmol of Ni_3_FeN/ Ni_3_Fe-350-2h catalyst under 25 bar of CO_2_ + H_2_ mixture (3:2 ratio) at 100 °C for 16 h. As expected, the addition of H_2_ had a positive effect on the formic acid formation, which was enhanced more than 20-fold (from 2.06 to 43.7 mM), and the generated formamide amount was more than doubled to 1.42 mM (Figure S19). Obviously, an increase in the formic acid amount promotes formamide formation. However, since nitrogen is the limiting reagent for the formation of formamide, the increase in concentration was much more significant for formate, suggesting that the reaction mechanism of CO_2_ to formamide is H_2_-dependent.

Additional experiments were performed using formic acid and NH_4_OH as substrates to gain more insights into the reaction pathway of the reductive CO_2_ conversion to formamide. Formic acid is commonly used as a substrate for amide synthesis.^21,23^ Moreover, a known method to prepare formamide is the reaction of formic acid and ammonia via ammonium formate formation as an intermediate product followed by dehydration to generate formamide at high temperatures.21 In order to investigate formic acid as a possible intermediate for formamide formation, we use 10 mM of formic acid as a carbon source (instead of CO_2_) since this concentration is close to the amount we have obtained in our reactions from CO_2_ reduction. The reaction was performed for 16 h under ambient conditions with the Ni_3_FeN/Ni_3_Fe-350-2h catalyst. ^1^H NMR result (Figure S20) showed that almost all formic acids were consumed, and 4.85 mM of formamide was calculated to be produced. In addition to formamide, 1.1 mM of acetamide was detected. This experiment indicates that formic acid is an intermediate product in formamide synthesis from CO_2_. After the formation of formic acid, the subsequent reaction with lattice nitrogen yields formamide as the final product. Dehydration of ammonium formate, a product of formic acid and ammonia, can form formamide and some metal nitrides were reported to yield ammonia in water.^68^ Therefore, it is possible that ammonia and formic acid are formed in water and yield formamide.

To reveal the role of nitrogen source, an additional experiment was conducted using 1 mM of NH_4_OH solution as a nitrogen source instead of nitride mineral. The reaction was performed with 0.5 mmol of pristine Ni_3_Fe nanoparticles (nitrogen-free) under 25 bar of CO_2_ at 100 °C for 16 h. This results in only a small amount of acetamide, and no formamide formation could be detected based on ^1^H NMR analyses (Figure S21). The reason might be the increase in the pH of the solution; alkaline pH does not promote the formation of formamide as mentioned above. This suggests a route of the direct consumption of lattice nitrogen in the Ni_3_FeN/Ni_3_Fe heterostructure to produce formamide instead of ammonia as an intermediate product.

**Figure 4:**
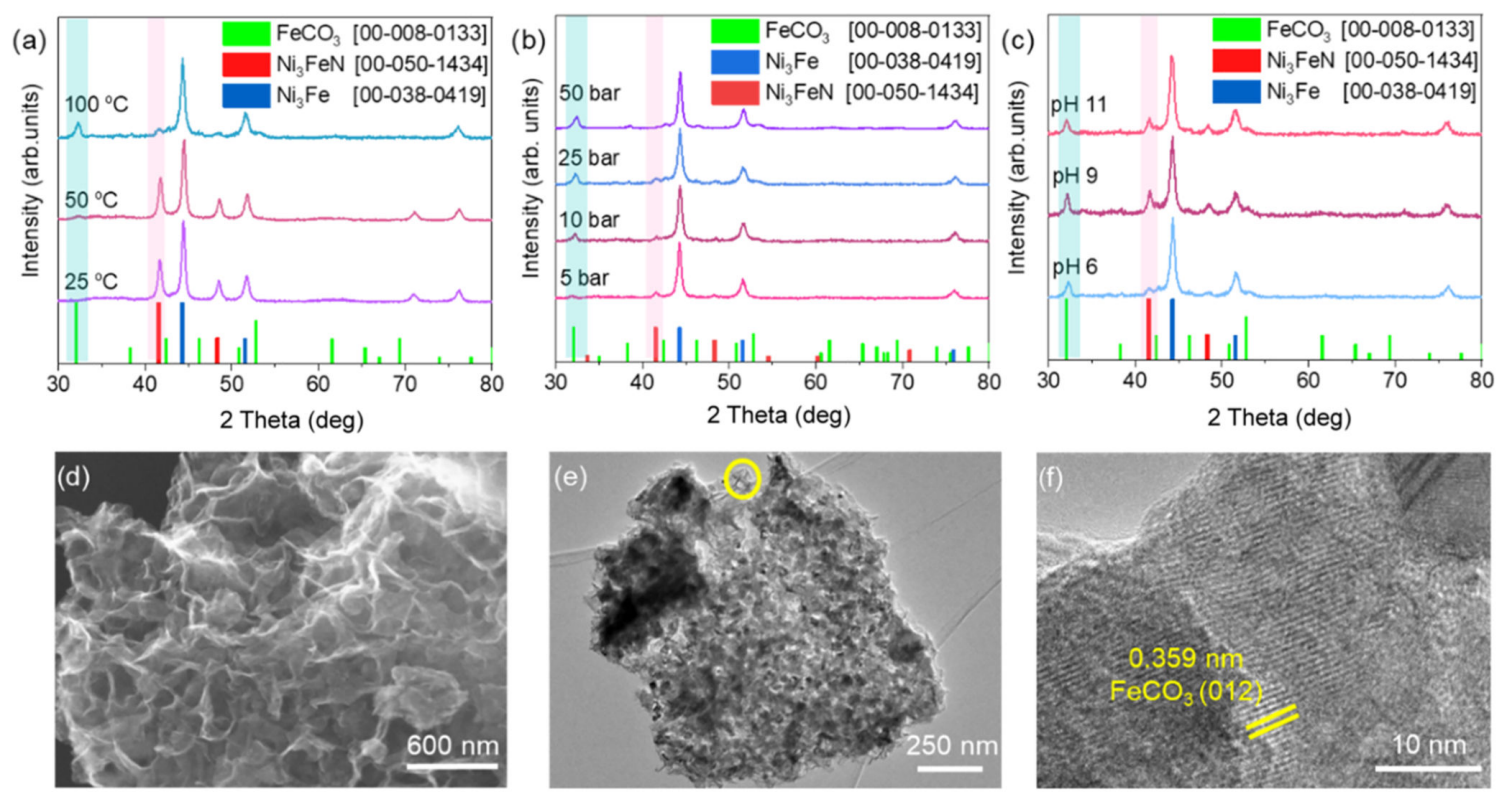
Postreaction XRD patterns of the Ni_3_FeN/Ni_3_Fe-350-2h catalyst at different reaction temperatures with 25 bar of CO_2_ (a), with different CO_2_ pressures at a reaction temperature of 100 °C (b), and at different initial pH values at 100 °C after 16 h of reaction time (c). SEM (d), TEM (e), and HR-TEM (f) images of the Ni_3_FeN/Ni_3_Fe-350-2h after the catalytic reaction under 25 bar of CO_2_ at pH 6 at 100 °C for 16 h.

**Figure 5:**
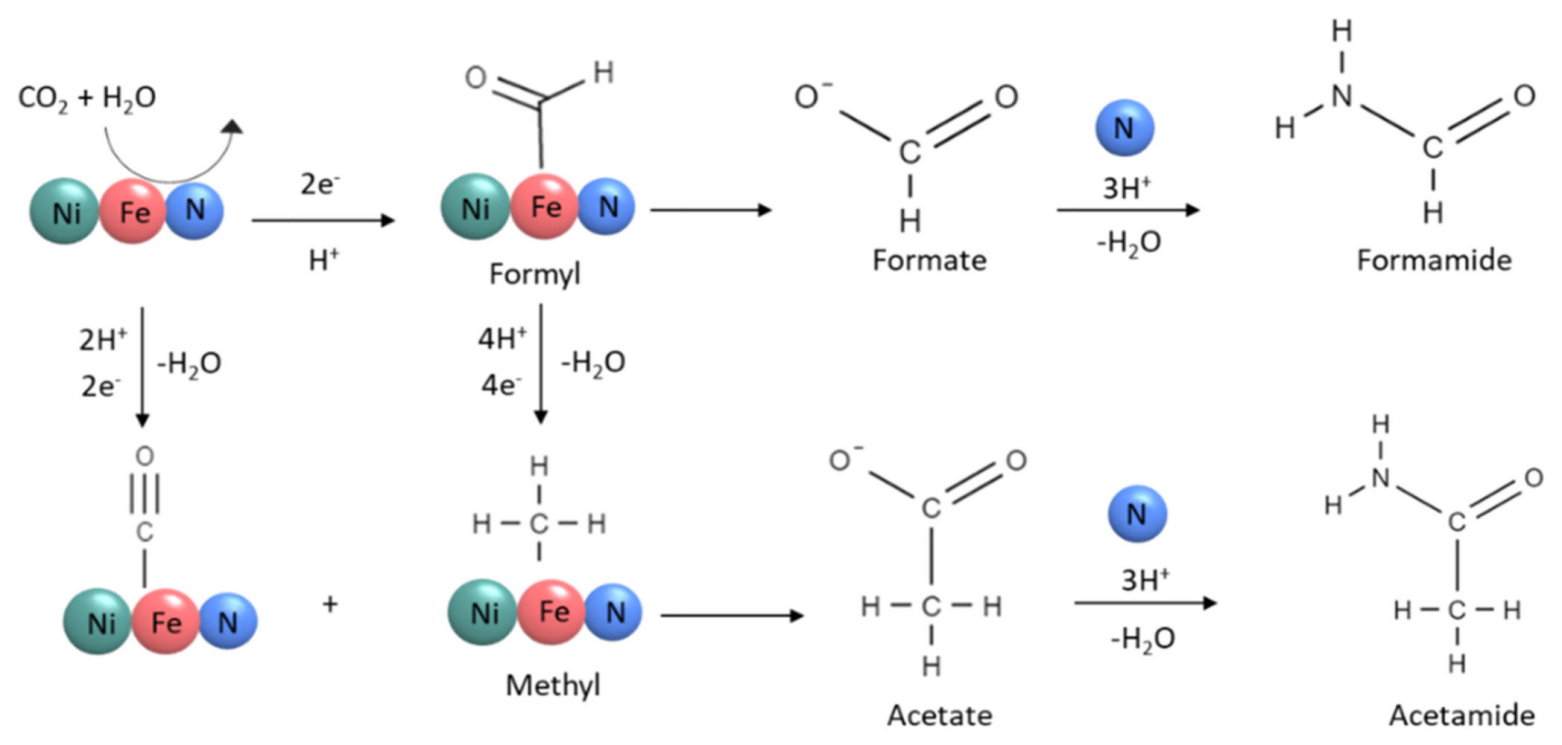
Possible reaction pathway for the formation of amides from CO_2_ and H_2_O over the Ni_3_FeN/Ni_3_Fe heterostructure.

It is important to study the alteration of the catalyst for a better understanding of the reaction mechanism. The selected Ni_3_FeN/Ni_3_Fe-350-2h powders were subjected to structural characterization after different catalytic reactions. The main results are depicted in Figure 4. As presented in XRD patterns in Figure 4a, the Ni_3_FeN/Ni_3_Fe phase has not been altered noticeably at reaction temperatures of 25 and 50 °C; however, it started to decompose, and the FeCO_3_ phase was formed at 100 °C. Postreaction SEM−EDX analyses indicated a clear compositional alteration after a reaction temperature of 25 and 100 °C. No nitrogen could be detected after the reaction at 100 °C (Figure S22), while about 5 atom % of nitrogen was found in the sample after the catalytic reaction temperature of 25 °C (Figure S23). EDX results also show that the Ni/Fe ratio was altered to be 5.5 and 4.4 after the reaction at 100 and 25 °C, respectively. This trend is expected due to the higher oxidation tendency of Fe compared to Ni in water. When the initial partial pressure of CO_2_ in the reaction was increased from 5 to 50 bar, the formation of the FeCO_3_ phase became more prominent as shown in XRD patterns in Figure 4b. At higher pressures, CO_2_ can be adsorbed on the catalyst surface more strongly and can cause its sudden deactivation due to the saturation of water with dissolved CO_2_. In addition, a higher amount of dissolved CO_2_ can decrease the pH of the reaction solution slightly. The oxidation of transition metals is promoted by acidic conditions. This can be seen from the postreaction XRD pattern of Ni_3_FeN/Ni_3_Fe-350-2h after the reactions at different pH values (Figure 4c). The Ni_3_FeN phase seems to be more durable under alkaline conditions of pH 9 and 11. Nitrides of Ni and Fe are reported to be less stable at acidic pH values.^69^ For instance, the stability of monometallic Ni_3_N highly depends on the pH. For pH values lower than 9, Ni_3_N can be oxidized to Ni_2_OH^3+^, while Ni_3_N is stable at pH values around 9.^69^ Besides reaction temperature, pressure, and pH, the reaction time was also found to affect catalyst alteration. As seen in the XRD pattern in Figure S24, FeCO_3_ formation was observed after 16, 24, and 72 h subsequent to the catalytic reaction with 25 bar of CO_2_ at 100 °C. The Ni_3_Fe phase could be detected even after 72 h, while no metal nitride phase could be observed after longer reaction times. Alteration of the morphology of a selected sample (Ni_3_FeN/Ni_3_Fe-350-2h) after the particular reaction conditions (25 bar of CO_2_ at 100 °C for 16 h) was further investigated by electron microscopy. SEM imaging shows significant alteration of the morphology and the formation of flake-like structures (Figure 4d). This structural alteration could be further supported by TEM where flake-like and aggregated nanoparticle structures were visualized (Figure 4e). As seen in Figure 4f, characteristic lattice fringes obtained from HR-TEM further support the formation of FeCO_3_ for the solid sample after the catalytic reaction under 25 bar of CO_2_ at 100 °C for 16 h. Overall, the formation of FeCO_3_ alters the morphology of the Ni_3_FeN/Ni_3_Fe-350-2h heterostructure. Surface alteration of the selected sample (Ni_3_FeN/Ni_3_Fe-350-2h) after the catalytic reaction was further examined by XPS. As displayed in Figure S25a, the Ni 2p spectrum contains a peak with maxima at 855.6 eV, which corresponds to Ni^2+^ species.^53^ Additionally, there is a small shoulder at 851.6 eV that can be assigned to the metallic Ni.^53^ The Fe 2p spectrum (Figure S25b) displays a peak located at 711.3 eV, which can be attributed to Fe^3+^.^70^ There was no detectable metallic Fe on the surface after the reaction. The surface Fe was completely oxidized, while the Ni surface was found to be more durable under the catalytic reaction conditions. No nitrogen species could be detected on the surface of this selected sample after the reaction, which further supports the consumption of nitrogen during the reaction. Alteration of monometallic Fe_*x*_N and Ni_3_N provides further hints about their catalytic performance in CO_2_ reduction. While the Ni_3_N phase was transferred to Ni during the catalytic CO_2_ reduction in the presence of water, the Fe_*x*_N catalyst was completely converted to the FeCO_3_ phase (Figure S26). Postreaction characterization of metal nanoparticles provided insights into the source of hydrogen and nitrogen for CO_2_ fixation. The formation of FeCO_3_ was observed after the catalytic reactions with Fe-containing catalysts. Interaction between carbonated water and Fe results in a redox reaction, which yields H_2_ via water decomposition and Fe oxidation. When the concentrations of CO_3_^2−^ and Fe^2+^ ions reach the solubility limit in the reaction solution, the precipitation of FeCO_3_ occurs. The precipitation of FeCO_3_ depends on several parameters including temperature, pH, and the partial pressure of CO_2_. At lower reaction temperatures, the dissolved CO_2_ amount in water is higher according to Henry’s law, but the kinetics of the FeCO_3_ formation are low. Therefore, the hydrogen formation rate is also low, which explains the lower concentrations of formate and formamide at lower reaction temperatures. In addition, the pH of the reaction affects the water dissociation rate over the metal catalyst. In alkaline regimes, the existence of OH^−^ causes a lower concentration of H^+^ in the solution, which could be the rate-limiting agent for CO_2_ fixation to formate. The reaction path for CO_2_ fixation to amides might follow a mechanism similar to Mars–van Krevelen, in which lattice nitrogen within nitrides involves C–N coupling and generation of nitrogen compounds.^71^ In the classical Mars–van Krevelen mechanism, after the adsorption of the substrate, an oxidation–reduction sequence occurs on the oxide surface whereby one of the lattice oxygens is consumed during the catalyst reduction step. In our reaction system, lattice nitrogen of Ni–Fe nitride heterostructures directly reacts with the CO_2_ and other intermediates. A simplified proposed reaction pathway for the formation of amides over Ni_3_FeN/Ni_3_Fe nanoparticles via direct CO_2_ reduction in H_2_O is shown in Figure 5. CO_2_ first can be converted to bonded CO or formyl group on the metal surface.59 The formyl group is either detached and forms formate or further converted to acetate. An amide formation pathway was confirmed by the reaction of formic acid and ammonia in our control experiment. Formic acid could be an intermediate during formamide formation. When the nitrogen source was replaced with ammonia, no formamide was obtained. Therefore, the coupling of *CHO and dissolved lattice N can generate formamide, which can occur even under ambient conditions. Furthermore, acetamide was observed in some reactions together with acetate. The formation of acetamide from acetate can follow a similar pathway to formamide, in which the −OH group is substituted with NH_2_.

## Conclusions

We have shown that nickel–iron nitride heterostructures can act as catalysts and substrates to convert CO_2_ and water to oxygenates and amides under mild hydrothermal conditions, without using any synthetic hydrogen and nitrogen. While monometallic Ni and Fe nitrides did not yield any formamide, bimetallic Ni−Fe nitride heterostructures yield formate, formamide, acetate, and acetamide. The formation of formate and amides was found to be very sensitive to the reaction conditions including temperature, pressure, pH, and reaction time. Postcharacterization analyses indicated the alteration of catalyst, dissolution of nitrogen from the lattice structure, and formation of metal carbonate phase. The outcome of this study showed that CO_2_ and water could be fixed to formamide, which is an important building block for the synthesis of prebiotic organics. Since the direct incorporation of N_2_ gas into the carbon fixation system is demanding, using a metal-based solid catalyst with chemisorbed nitrogen for the direct synthesis of formamide from CO_2_ and H_2_O can provide a different perspective for the possible formation scenario of formamide under hydrothermal vent conditions.

## Experimental Methods

### Reagents and Materials

Fe(NO_3_)_3_·9H_2_O (≥98%) and Ni-(NO_3_)_2_·6H_2_O (≥97%) and all of the standards were obtained from Merck. Tea leaves were purchased from Goran-Tee.

### Synthesis of Nickel−Iron Nitrides

Nickel−Iron oxide nan-oparticles were prepared through a hard-templating route by using spent tea leaves as a carbon-based template.^72^ Briefly, tea leaves were washed with distilled H_2_O at 80 °C several times and dried at 80 °C overnight. For the synthesis of NiO, 0.1 M of aqueous solution of Ni(NO_3_)_2_·6H_2_O was prepared and dried tea leaves were added to this solution. The mass ratio of tea leaves to total metal precursor was adjusted to 2:1. After continuously stirring at room temperature for 2 h, the tea leaves were dried at 80 °C overnight and calcined at 550 °C (with a heating rate of 2 °C/min) under air for 4 h. For the synthesis of Fe_2_O_3_, the same procedure was implemented by using an aqueous solution of Fe(NO_3_)_3_·9H_2_O. Ni_3_Fe nanoparticles were prepared by setting the molar ratio between Ni and Fe salt precursors to 3:1. After the drying process at 80 °C, the composite material was calcined at 550 °C for 4 h to remove the carbon and obtain metal oxide nanoparticles (ramping rate is 2 °C/min). The diluted acid leaching method was used to remove possible residues, such as Ca and Mg, after the calcination of spent tea leaves. For that, the synthesized metal oxides were washed with 40 mL of 0.1 M HCl for 2 h (4 h for Fe) and centrifuged three times with H_2_O. Upon acid treatment and leaching, samples were dried at 80 °C overnight. The reduction of synthesized metal oxides was performed with 10% H_2_/Ar gas flow (total flow rate: 100 mL/min) at 500 °C for 2 h to obtain reduced metal nanoparticles (ramping rate is 2 °C/min). In order to prevent the complete oxidation of metal nanoparticles after H_2_ reduction, the surface passivation process with air/Ar gas flow (100 mL/min, 2% air) was performed at room temperature for 1 h.^38^

Ni−Fe nitrides were obtained via the ammonolysis method. After H_2_ reduction, metal powders were subjected to ammonia treatment in the tube furnace. The samples were prepared according to the following procedures: the Ni_3_FeN/Ni_3_Fe heterostructure was prepared by the reaction of reduced Ni_3_Fe and ammonia in a tube furnace at the temperature range of 300−400 °C for 1 or 2 h. The heating rate was 10 °C/min, and the flow rate of ammonia gas was 100 mL/min. The samples were labeled as follows:

Ni_3_Fe sample treated at 350 °C for 2 h: Ni_3_FeN/Ni_3_Fe-350-2h, Ni_3_Fe sample treated at 300 °C for 2 h: Ni_3_FeN/Ni_3_Fe-300-2h, Ni_3_Fe sample treated at 350 °C for 2 h: Ni_3_FeN/Ni_3_Fe-350-1h, Ni_3_Fe sample treated at 300 °C for 2 h: Ni_3_FeN/Ni_3_Fe-300-2h.

Ni_3_N/Ni sample was prepared by treating metallic Ni with an ammonia flow at 300 °C for 2 h with a flow rate of 100 mL/min. The Fe_*x*_N/Fe heterostructure was obtained by treating metallic Fe with ammonia at 350 °C for 2 h. After ammonia treatment, the furnace was cooled down under ammonia flow to prevent the decomposition of nitrides at high temperatures. Upon reaching room temperature, the system was purged with an Ar flow (100 mL/min) for 2 h and the samples were left in the quartz tube overnight under ambient conditions in order to naturally passivate the surface of nitride samples in order to prevent the exchange of nitrogen with oxygen when the samples interact with air.

### Structural Characterization

Crystal structures of synthesized materials were analyzed by powder X-ray diffraction (XRD) using Stoe theta/theta diffractometer with the Bragg−Brentano geometry using Cu K*α*1/2 radiation. In situ high-temperature X-ray diffraction data were collected on a Rigaku SmartLab with a rotating anode (9 kW, 45 kV, 200 mA) in the Bragg−Brentano geometry (Cu K*α*1/2: 1.541862 Å). Data were collected with a HyPix-3000 multidimensional detector in 1D mode. A reaction chamber (XRK900, Anton Paar) was mounted on the diffractometer for the heating experiments. Heating was performed from room temperature to 30 °C and from 200 to 400 °C (10 K/min) in 20 °C steps where the sample was kept for 30 min each under a constant flow of 20 mL/min NH_3_. Data were collected continuously in the range of 35−80° 2*θ* in steps of 0.01° and a scan speed of 6°/min. For each temperature, three scans were collected and then merged. N_2_-sorption analysis was used to determine the textural parameters of synthesized Ni−Fe nitride heterostructures. N_2_-physisorption isotherms were measured with a 3Flex Micromeritics setup at −196 °C. Before each measurement, samples were degassed at 150 °C for 10 h. Specific surface areas were determined by applying the Brunauer−Emmett−Teller (BET) method in the relative pressure range between 0.06 and 0.2. The morphology of samples was investigated by the transmission electron microscopy (TEM) imaging of powder samples using a Hitachi H-7100 (100 kV). Lattice fringes are obtained with high-resolution TEM micrographs collected with a Hitachi HF2000. Scanning electron microscopy/scanning transmission electron microscopy−energy-dispersive X-ray spectroscopy (SEM/STEM−EDX) mapping was performed with a Hitachi S-3500N electron microscope. The alteration of the catalyst after the reaction was analyzed by XRD. The postreaction catalyst was washed with 40 mL of distilled water and dried in the vacuum furnace at 50 °C prior to the measurement. Dry powder was directly measured with XRD.

### CO_2_ Reduction Experiments

CO_2_ reduction reactions were performed by using an in-house built autoclave made of Mo−Ni alloy, which provides stability for high-pressure and -temperature conditions. Poly(tetrafluoroethylene) (PTFE) inlet with a volume of 28 mL was utilized in order to prevent possible contaminations and catalytic effects coming from the metallic reactor. The reaction temperature and pressure were monitored by the thermocouple and the pressure transmitter, respectively. For a typical reaction, the reactor was loaded with 2 mmol (1 M) of the metal catalyst in 2 mL of H_2_O. Therefore, 0.5 mmol of Ni_3_FeN/Ni_3_Fe, 2 mmol of Ni_*x*_N/Ni, and Fe_*x*_N/Fe were used. Then, the reactor was pressurized with 25 bar of CO_2_ gas at different reaction temperatures (25−100 °C) and different initial pH values in the range of 6−11. For high-pressure reactions, the reactor was filled with 50 or 100 bar of CO_2_ and reactions were conducted at 100 °C for 16 h. The pH of alkaline reactions was adjusted by the addition of KOH solution (0.1 M) and verified with pH indicator strips (Merck, 1.09526.0003, Universal indicator, 376). Reactions under alkaline conditions were performed with 25 bar CO_2_ at 100 °C for 16 h.

Several control reactions were performed. First, the possible catalytic effect of the reactor was checked in the absence of the metal catalyst with 25 bar of CO_2_ in H_2_O at 100 °C. Furthermore, possible contamination from the catalyst (selected sample: Ni_3_FeN/Ni_3_Fe-350-2h) was checked by performing a reaction under 25 bar of Ar at 100 °C for 16 h. Additional experiments were performed by using either formic acid as a carbon source or NH4OH as a nitrogen source. For the formic acid conversion reaction, 10 mM formic acid solution was used as a carbon substrate with the Ni_3_FeN/Ni_3_Fe-350-2h heterostructure as a nitrogen source. After purging with Ar, the reaction was performed at 25 °C for 16 h. Then, the nitrogen source was changed to NH_4_OH solution and CO_2_ gas was used as a carbon source. For the ammonia reaction, 0.5 mmol of Ni_3_Fe metal was used with 1 mM of NH4OH solution under 25 bar of CO_2_ at 100 °C for 16 h.

After the reaction, the reactor was cooled down to room temperature for 2 h. The solid catalyst was removed after the reaction by centrifugation at 9000 rpm for 10 min. The liquid was then filtrated with a syringe and a filter (MULTOCLEAR-13 PTFE supplied by Chromatographie Service GmbH) with a 0.2 μm pore size in order to minimize possible adverse effects of metal particles on the liquid product analysis.

### Analyses of Products

For liquid product analysis, proton nuclear magnetic resonance (^1^H NMR) and high-performance liquid chromatography (HPLC) techniques were utilized. Standards of expected products including formic acid (≥98%, ACS reagent), sodium acetate (>99%), and sodium pyruvate (≥99%) were purchased from Merck and analyzed by HPLC and NMR before the experiments. Before each ^1^H NMR measurement, liquid samples were treated with 0.01 M of K_3_PO_4_ solution and centrifuged at 13 500 rpm for 15 min to minimize the paramagnetic effect coming from possibly leached metal species as it is described in our previous study.^67^

NMR spectra were obtained on either a Bruker Avance Neo spectrometer operating at a field of 14.1 T (^1^H Larmor frequency of 600 MHz) with a cryogenically cooled TCI probe for the highest sensitivity on the direct observation of ^1^H. All spectra were collected at 25 °C in standard 5 mm tubes containing sample volumes of about 700 *μ*L with the addition of 10% D_2_O (70 *μ*L) as it was described previously.^38,67^ In ^1^H spectra, water suppression at 4.68 ppm was achieved using “excitation sculpting” together with “perfect echo”using the Bruker standard pulse program “zgesgppe”. Concentrations of amides (formamide and acetamide) were calculated with ^1^H NMR by using pentaerythritol (100 *μ*M) as an internal standard.

A Shimadzu LC-2030 equipped with a refractive index (RI) detector was used for HPLC measurements. The column was Metacarb 67H with a 6.5 mm inner diameter and 300 mm length. The mobile phase is 0.1% of trifluoroacetic acid (TFA) at a flow rate of 0.8 mL/min, and the temperature was constant at 50 °C during the measurements. HPLC was used for the analysis of carboxylic acids including formic acid and acetic acid.

## Supplementary Material

Figures S1–S26
