# Supplementary material for: Direct Synthesis of Formamide from CO2 and H2O with Nickel–Iron Nitride Heterostructures under Mild Hydrothermal Conditions
Source: J Am Chem Soc. 2023 Aug 29;145(36):19768–79. doi: 10.1021/jacs.3c05412 (PMC7615090; doi:10.1021/jacs.3c05412)
Supplement: Supplementary file 1 — ja3c05412_si_001.pdf [file ja3c05412_si_001.pdf]

SUPPORTING INFORMATION

Direct Synthesis of Formamide from CO_2 and H_2O with Nickel-Iron Nitride Heterostructures under Mild Hydrothermal Conditions

Tuğçe Beyazay[1], William F. Martin[2], Harun Tüysüz[1*]

[1] Max-Planck-Institute für Kohlenforschung, Kaiser-Wilhelm-Platz 1, 45470, Mülheim an der Ruhr, Germany

[2] Institute of Molecular Evolution, University of Düsseldorf, 40225 Düsseldorf, Germany

*E-mail: tueysuez@kofo.mpg.de

Table of Figures

Figure S1. In-situ wide-angle powder XRD patterns of Ni_3Fe and Ni_3FeN formation under ammonia gas in the temperature range of 30-400 ˚C. 3
Figure S2. Wide-angle powder XRD patterns of Ni_3FeN/Ni_3Fe synthesized at 300 ˚C and 350 ˚C during 1 and 2 h of ammonia treatment. 3
Figure S3. N_2-sorption isotherms of Ni_3Fe before and after ammonia treatment at 300 and 350 ˚C for *2* h. 4
Figure S4. SEM-EDX elemental mapping of Ni_3FeN/Ni_3Fe-350-2h (a) and Ni_3FeN/Ni_3Fe-300-2h (b) 4
Figure S5. SEM-EDX elemental mapping of Ni_3FeN/Ni_3Fe-350-2h in 100 nm range. 4
Figure S6. Particle size distribution and average particle size of Ni_3FeN/Ni_3Fe-350-2h determined from TEM micrographs and number of counted particles: 139. 5
Figure S7. STEM-EDX line scanning of Ni_3FeN/Ni_3Fe-350-2h in order to show distribution of N, Ni, Fe atoms on nanoparticles. 5
Figure S8. XRD patterns of Ni_3N/Ni (Ni_3N PDF: 00-010-0280 and Ni PDF: 03-065-2865) (a), and Fe_xN/Fe (Fe_4N PDF: 00-006-0627, Fe_3N PDF: 00-049-1662, and Fe PDF: 00-006-0696) (b) after the treatment under ammonia gas 6
Figure S9. Images of the autoclave reactor (made of Mo-Ni alloy) and the PTFE inlet with a volume of 28 ml. 6
Figure S10. ^{1}H-NMR spectra of standard solutions of formamide (fd1) and acetamide (acd1) with the internal standard pentaerythritol (Pet1) 7
Figure S11. ^{1}H-NMR spectra of standards of possible carbon fixation products 8
Figure S12. ^{1}H-NMR spectrum of control reaction of Ni_3FeN/Ni_3Fe catalyst under 25 bar of Ar at 100 ˚C for 16 h (a), HPLC result of control reaction of the reactor in the absence of the catalyst (b). Ft: formate. 8
Figure S13. ^{1}H-NMR spectra of the reactions performed with Ni_3FeN/Ni_3Fe-300-2h and Ni_3FeN/Ni_3Fe-350-2h (a), zoomed area in formate and formamide region (b), comparison of product concentrations (c) under 25 bar of CO_2 at 100 ˚C for *16 h*. Amide concentrations are calculated from H-NMR with an internal standard (100 mM pentaerythritol), formate and acetate are calculated by HPLC. ft: formate, fd: formamide, pet: pentaerythritol, me: methanol, actn: acetone, acd: acetamide, act: acetate. Error bars are refer to standard deviations of three independent reactions. Formate region is highlighted in pink and formamide region is highlighted in green. 9
Figure S14. ^{1}H-NMR spectrum (a) and HPLC result (b) of the reaction performed with Fe_xN/Fe nanoparticles under 25 bar of CO_2 at 100 ˚C for 16 h. ft: formate, me: methanol, actn: acetone, act: acetate, pyr: pyruvate 9
Figure S15. Whole ^{1}H-NMR spectrum (a), zoomed area in the region of acetate and acetamide (b), and HPLC result (c) of the reaction with Ni_3N/Ni nanoparticles under 25 bar of CO_2 at 100 ˚C for 16 h. Acetamide concentration is 0.19 mM according to H-NMR. Ft: formate, Et: ethanol, actn: acetone, act: acetate, acd: acetamide. 10
Figure S16. Whole (a) and zoomed (b) ^{1}H-NMR spectra of the reactions with different amounts of Ni_3FeN/Ni_3Fe-350-2h under 25 bar of CO_2 at 100 ˚C for 16h. ft: formate, fd: formamide, me: methanol, actn: acetone, act: acetate, acd: acetamide. Acetamide and acetate are highlighted in purple and blue color, respectively. 10
Figure S17. Whole (a) and zoomed (b) ^{1}H-NMR spectra of reactions performed over Ni_3FeN/Ni_3Fe-350-2h particles under 25 bar of CO_2 at 100 ˚C for 16 h, 24h, 72 h, and 168 h. ft: formate, fd: formamide, actn: acetone, act: acetate, acd: acetamide. 11
Figure S18. Gas products of the reactions performed with Ni_3FeN/Ni_3Fe-350-2h under 25 bar of CO_2 at 100 ˚C for 16 h (a) and 72 h (b). 11
Figure S19. ^{1}H-NMR spectrum (a), and product concentrations (b) of the reaction performed with 25 bar $CO_2 + H_2$ mixture (3:2 ratio) at 100 ˚C for 16 h over Ni_3FeN/Ni_3Fe-350-2h heterostructure. 12
Figure S20. ^{1}H-NMR spectrum of formic acid (10 mM) conversion reaction at room temperature over Ni_3FeN/Ni_3Fe-350-2h particles for 16 h. ft: formate, fd: formamide, dmc: dimethylcarbonate, me: methanol, actn: acetone, acd: acetamide, act: acetate 12
Figure S21. ^{1}H-NMR spectrum of the reaction performed with NH_4OH solution (1 mM) with Ni_3FeN/Ni_3Fe-350-2h nanoparticles under 25 bar of CO_2 at 100 ˚C for 16 h. 13
Figure S22. SEM-EDX results of Ni_3FeN/Ni_3Fe-350-2h sample after the reaction with 25 bar CO_2 at pH 6 and 100 ˚C for 16 h 13
Figure S23. SEM-EDX results of Ni_3FeN/Ni_3Fe-350-2h sample after the reaction with 25 bar CO_2 at pH 6 and 25 ˚C for 16 h 14
Figure S24. Post-reaction XRD patterns of Ni_3FeN/Ni_3Fe-350-2h after 16 h, 24 h, and 72 h with 25 bar of CO_2 at 100 ˚C. . 14
Figure S25. High-resolution XPS spectra of Ni 2p and Fe 2p of Ni_3FeN/Ni_3Fe-350-2h heterostructure after the reaction under 25 bar of CO_2 at 100 ˚C for 16 h. 15
Figure S26. XRD patterns of Ni_3N/Ni (a) and Fe_xN (b) after the reaction under 25 bar of CO_2 at 100 ˚C for 16 h. Ni_3N PDF: 00-010-0280, Ni PDF: 03-065-2865, Fe_4N PDF: 00-006-0627, Fe_3N PDF: 00-049-1662, Fe PDF: 00-006-0696, $FeCO_3$ PDF: 00-038-0419. 15

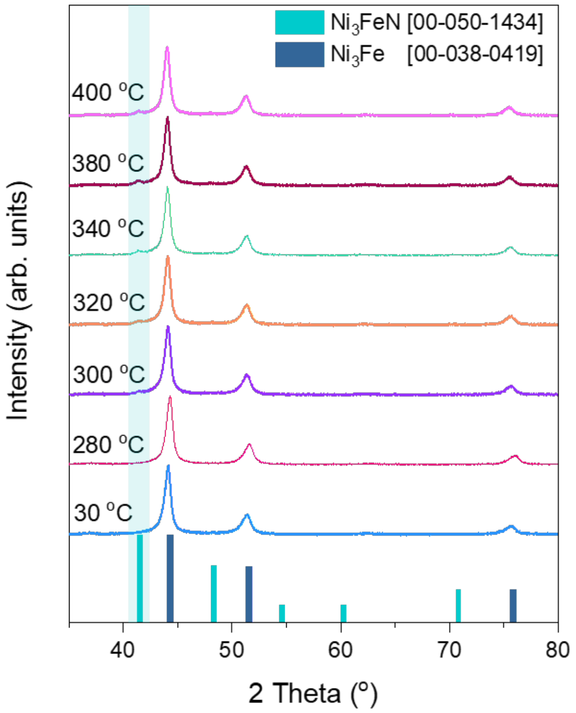

Figure S1. In-situ wide-angle powder XRD patterns of Ni_3Fe and Ni_3FeN formation under ammonia gas in the temperature range of 30-400 ˚C.

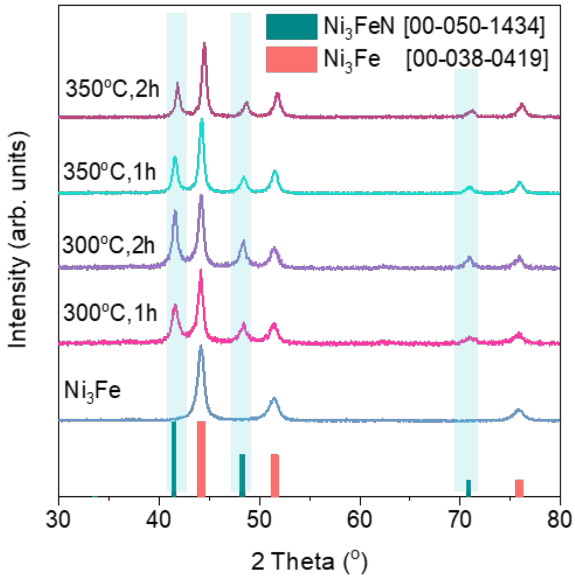

Figure S2. Wide-angle powder XRD patterns of Ni_3FeN/Ni_3Fe synthesized at 300 ˚C and 350 ˚C during 1 and 2 h of ammonia treatment.

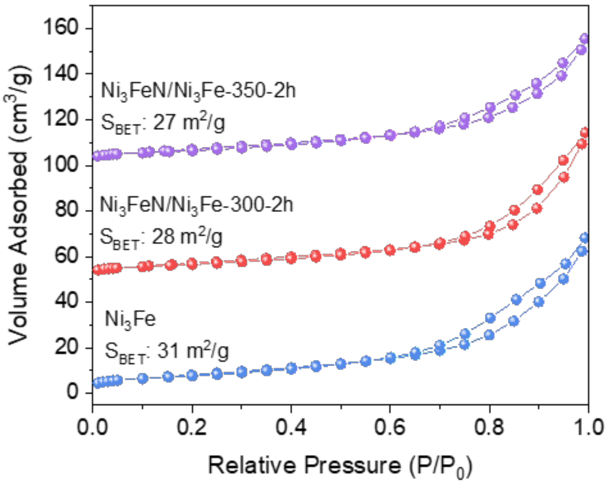

Figure S3. N_2-sorption isotherms of Ni_3Fe before and after ammonia treatment at 300 and 350 ˚C for *2* h.

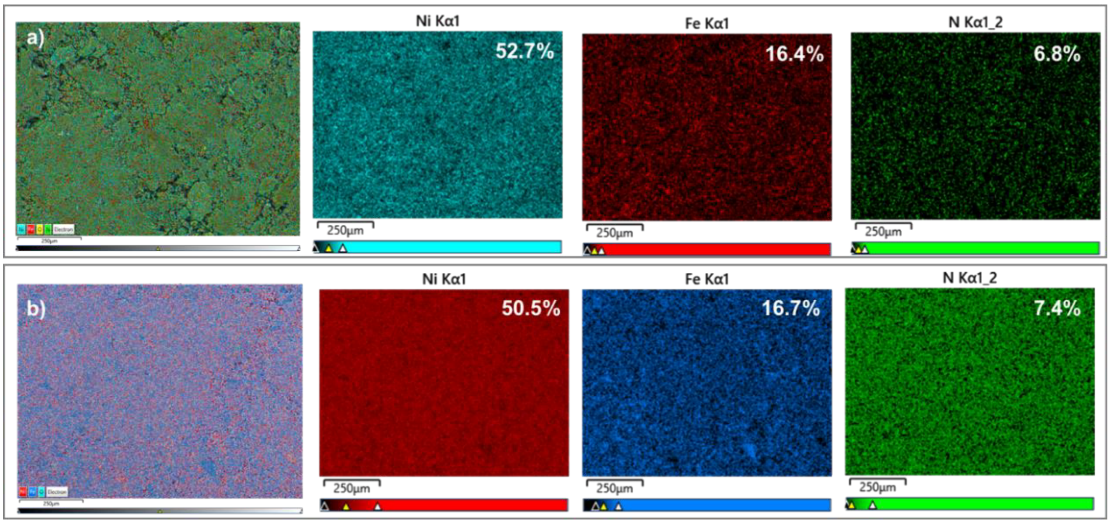

Figure S4. SEM-EDX elemental mapping of Ni_3FeN/Ni_3Fe-350-2h (a) and Ni_3FeN/Ni_3Fe-300-2h (b).

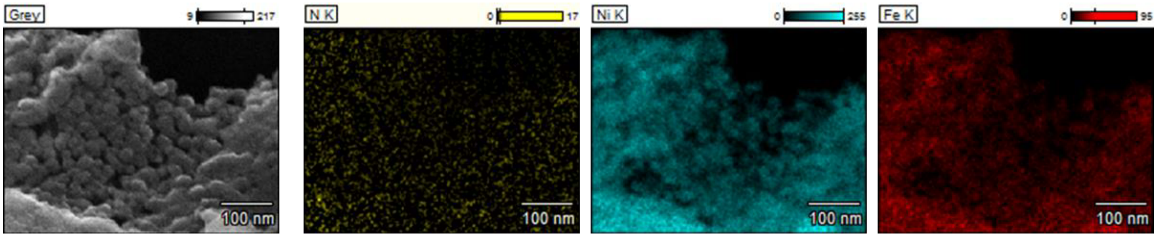

Figure S5. SEM-EDX elemental mapping of Ni_3FeN/Ni_3Fe-350-2h in 100 nm range.

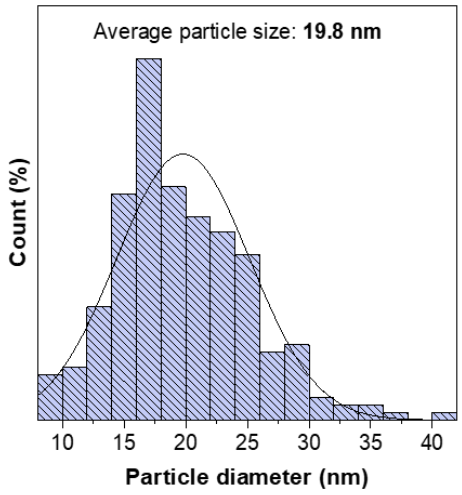

Figure S6. Particle size distribution and average particle size of Ni_3FeN/Ni_3Fe-350-2h determined from TEM micrographs and number of counted particles: 139.

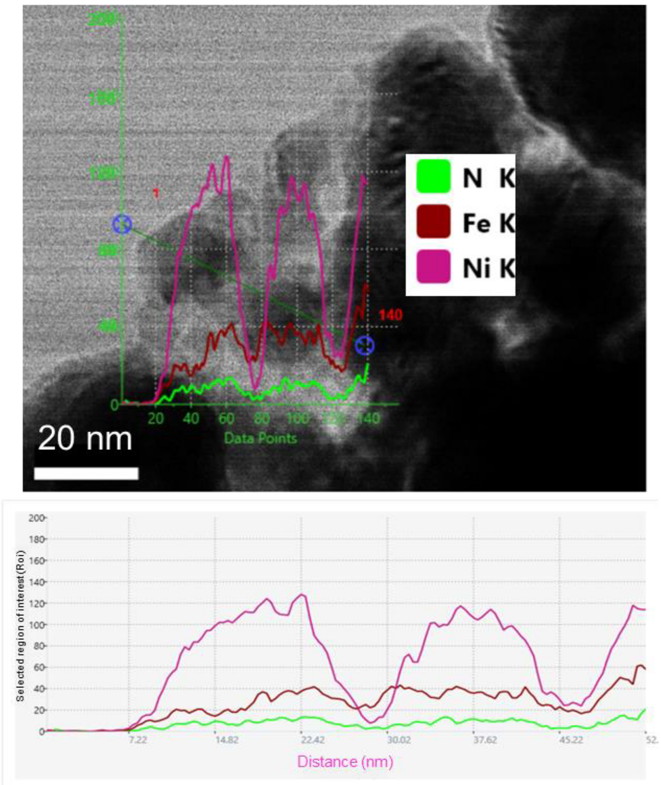

Figure S7. STEM-EDX line scanning of Ni_3FeN/Ni_3Fe-350-2h in order to show distribution of N, Ni, Fe atoms on nanoparticles.

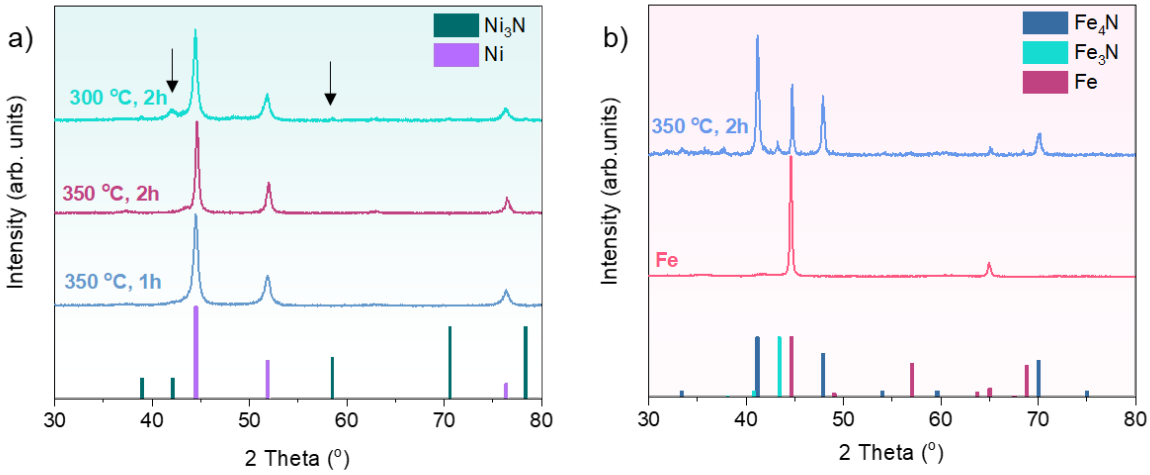

Figure S8. XRD patterns of Ni_3N/Ni (Ni_3N PDF: 00-010-0280 and Ni PDF: 03-065-2865) (a), and Fe_xN/Fe (Fe_4N PDF: 00-006-0627, Fe_3N PDF: 00-049-1662, and Fe PDF: 00-006-0696) (b) after the treatment under ammonia gas.

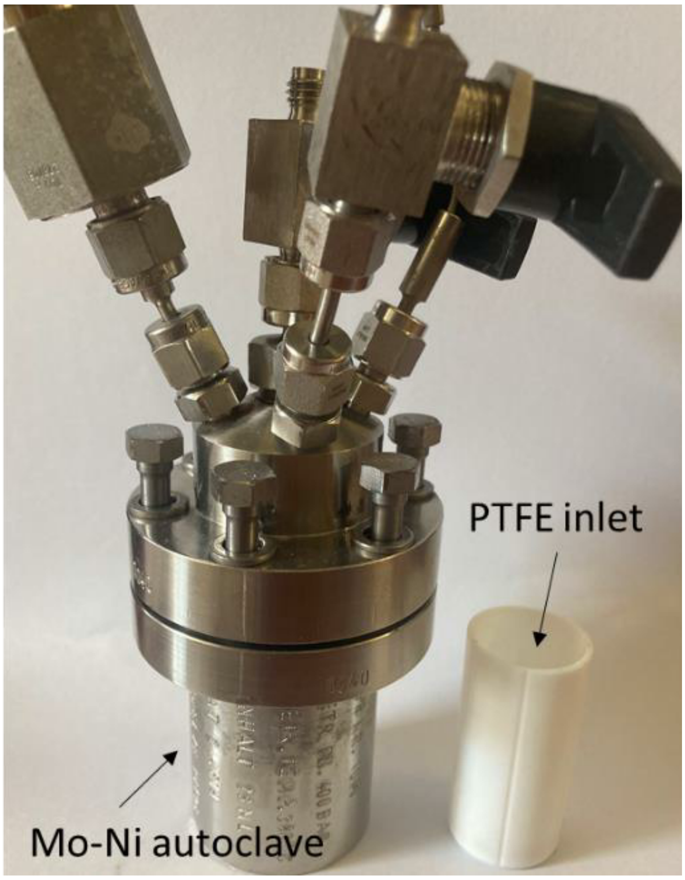

Figure S9. Images of the autoclave reactor (made of Mo-Ni alloy) and the PTFE inlet with a volume of 28 ml.

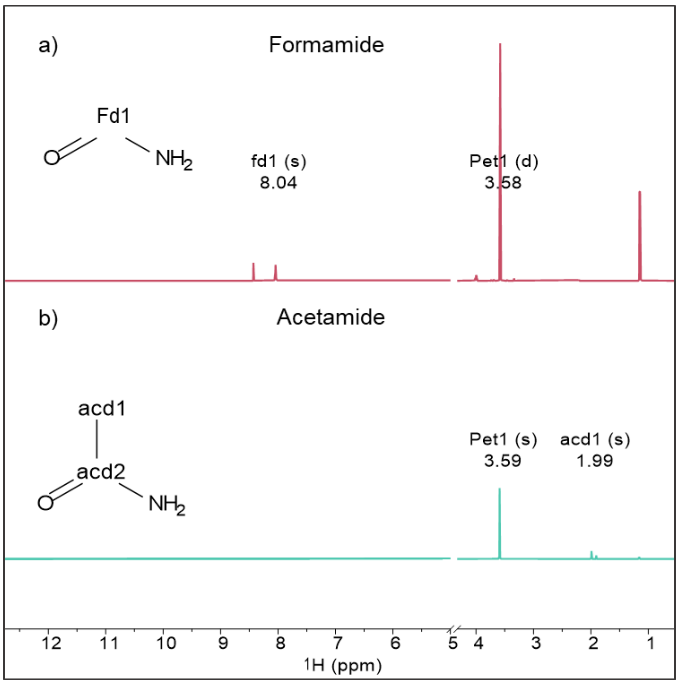

Figure S10. ^{1}H-NMR spectra of standard solutions of formamide (fd1) and acetamide (acd1) with the internal standard pentaerythritol (Pet1).

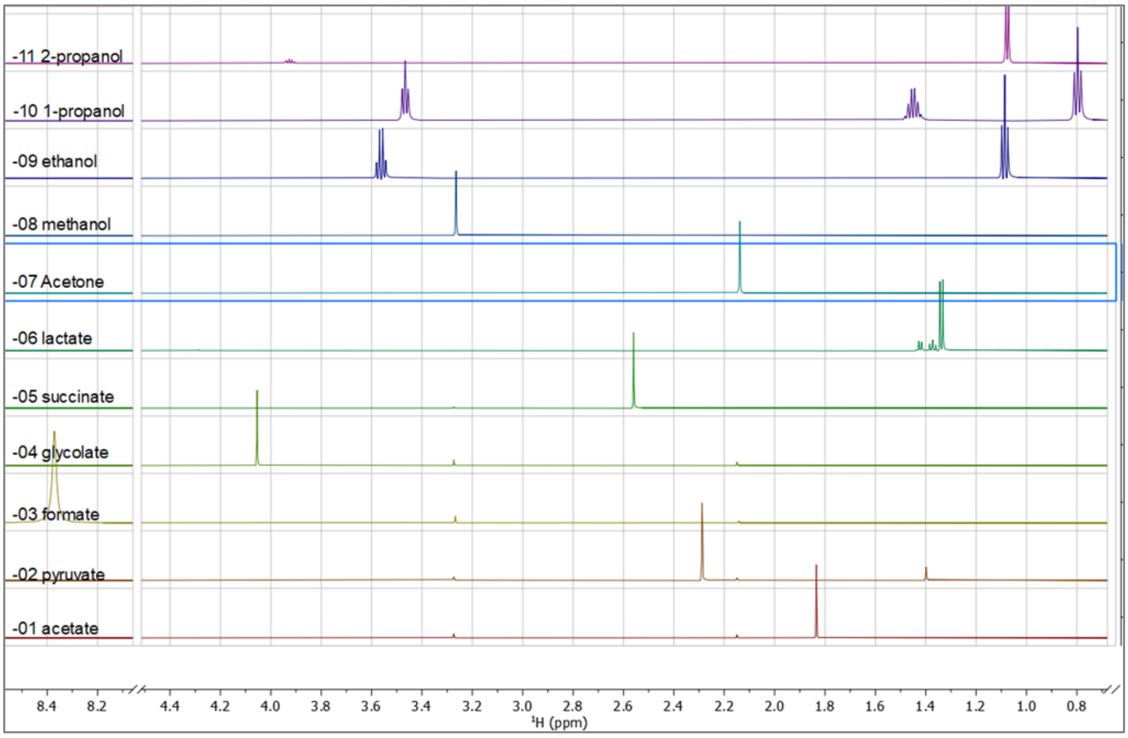

Figure S11. ^{1}H-NMR spectra of standards of possible carbon fixation products.

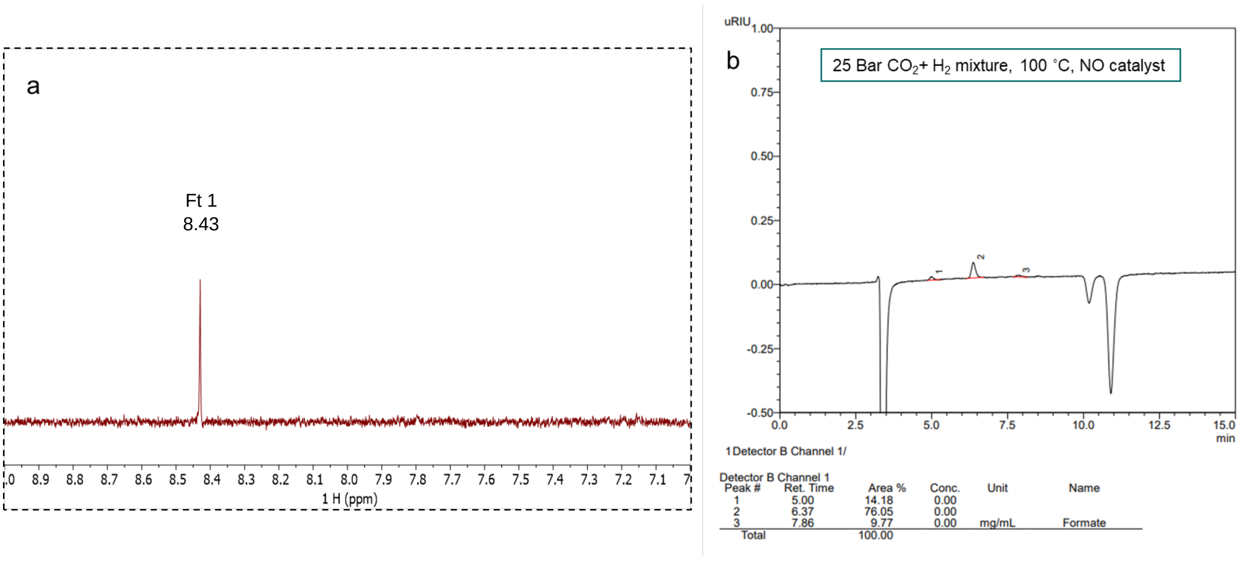

Peak #	Ret. Time	Area %	Conc.	Unit	Name
1	5.00	14.18	0.00		
2	6.37	76.05	0.00		
3	7.86	9.77	0.00	mg/mL	Formate
Total		100.00			

Figure S12. ^{1}H-NMR spectrum of control reaction of Ni_3FeN/Ni_3Fe catalyst under 25 bar of Ar at 100 ˚C for 16 h (a), HPLC result of control reaction of the reactor in the absence of the catalyst (b). Ft: formate.

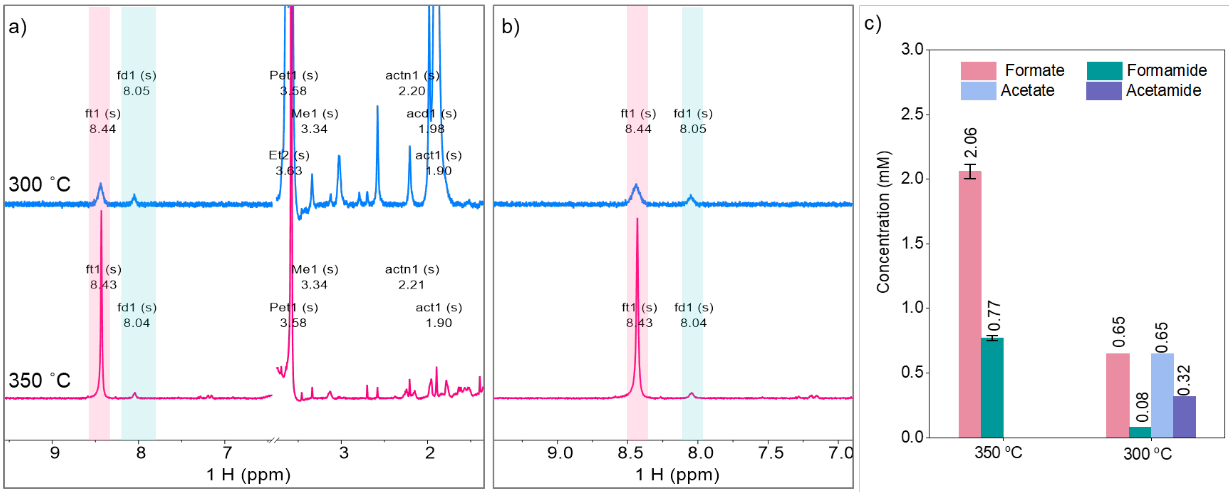

Figure S13. ^{1}H-NMR spectra of the reactions performed with Ni_3FeN/Ni_3Fe-300-2h and Ni_3FeN/Ni_3Fe-350-2h (a), zoomed area in formate and formamide region (b), comparison of product concentrations (c) under 25 bar of CO_2 at 100 ˚C for *16 h*. Amide concentrations are calculated from H-NMR with an internal standard (100 mM pentaerythritol), formate and acetate are calculated by HPLC. ft: formate, fd: formamide, pet: pentaerythritol, me: methanol, actn: acetone, acd: acetamide, act: acetate. Error bars are refer to standard deviations of three independent reactions. Formate region is highlighted in pink and formamide region is highlighted in green.

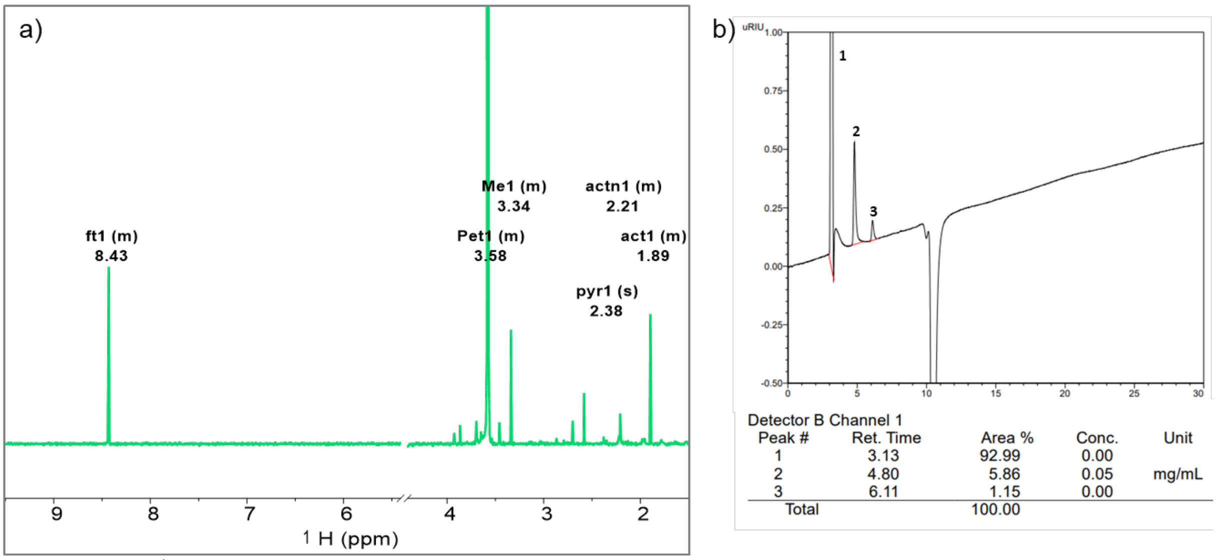

Figure S14. ^{1}H-NMR spectrum (a) and HPLC result (b) of the reaction performed with Fe_xN/Fe nanoparticles under 25 bar of CO_2 at 100 ˚C for 16 h. ft: formate, me: methanol, actn: acetone, act: acetate, pyr: pyruvate.

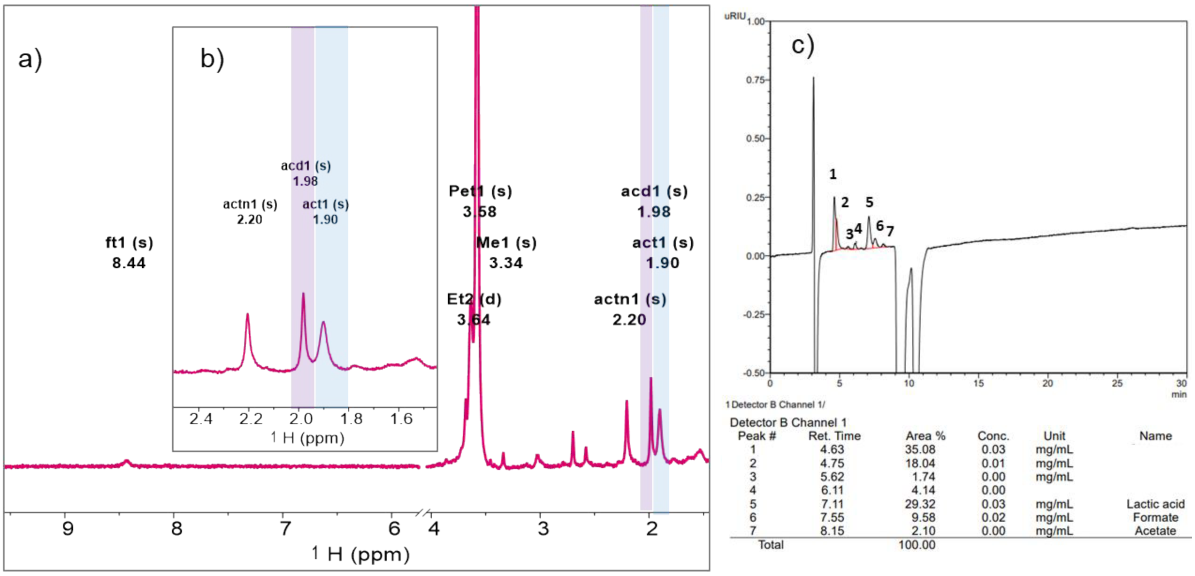

Peak #	Ret. Time	Area %	Conc.	Unit	Name
1	4.63	35.08	0.03	mg/mL	
2	4.75	18.04	0.01	mg/mL	
3	5.62	1.74	0.00	mg/mL	
4	6.11	4.14	0.00		
5	7.11	29.32	0.03	mg/mL	Lactic acid
6	7.55	9.58	0.02	mg/mL	Formate
7	8.15	2.10	0.00	mg/mL	Acetate
Total		100.00			

Figure S15. Whole ^{1}H-NMR spectrum (a), zoomed area in the region of acetate and acetamide (b), and HPLC result (c) of the reaction with Ni_3N/Ni nanoparticles under 25 bar of CO_2 at 100 ˚C for 16 h. Acetamide concentration is 0.19 mM according to H-NMR. Ft: formate, Et: ethanol, actn: acetone, act: acetate, acd: acetamide.

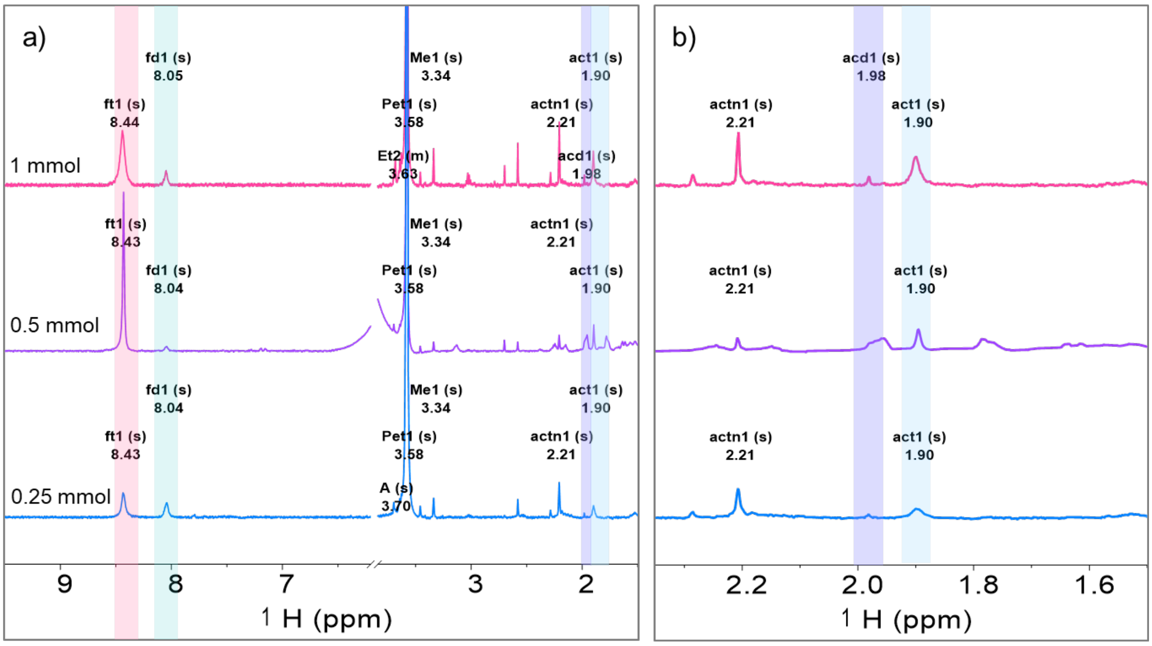

Figure S16. Whole (a) and zoomed (b) ^{1}H-NMR spectra of the reactions with different amounts of Ni_3FeN/Ni_3Fe-350-2h under 25 bar of CO_2 at 100 ˚C for 16h. ft: formate, fd: formamide, me: methanol, actn: acetone, act: acetate, acd: acetamide. Acetamide and acetate are highlighted in purple and blue color, respectively.

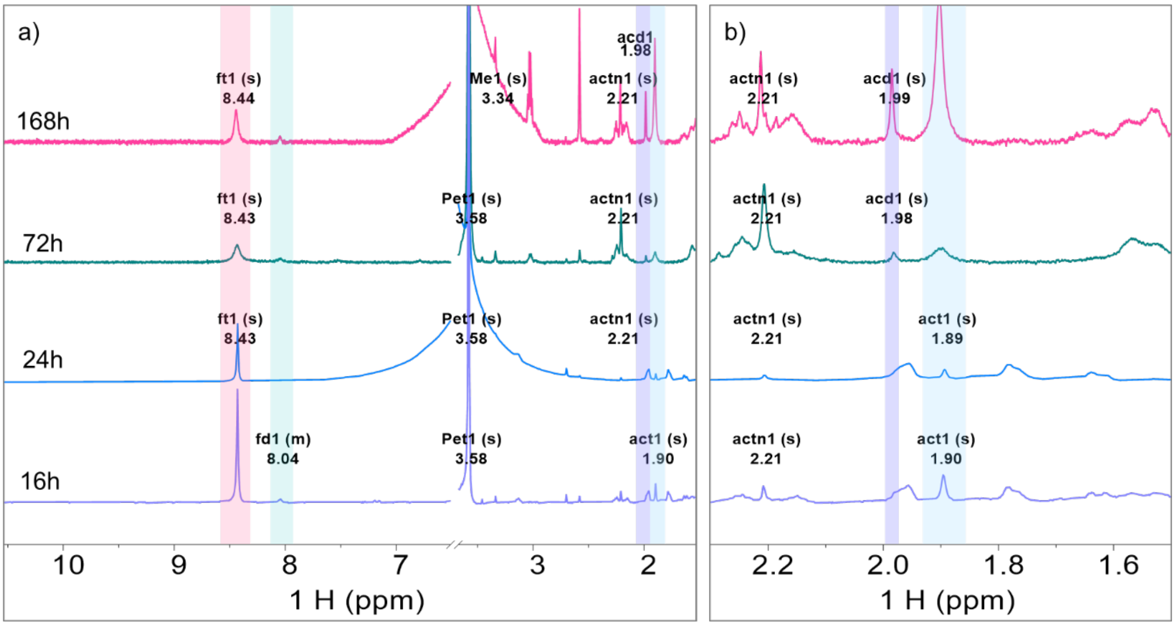

Figure S17. Whole (a) and zoomed (b) ^{1}H-NMR spectra of reactions performed over Ni_3FeN/Ni_3Fe-350-2h particles under 25 bar of CO_2 at 100 ˚C for 16 h, 24h, 72 h, and 168 h. ft: formate, fd: formamide, actn: acetone, act: acetate, acd: acetamide.

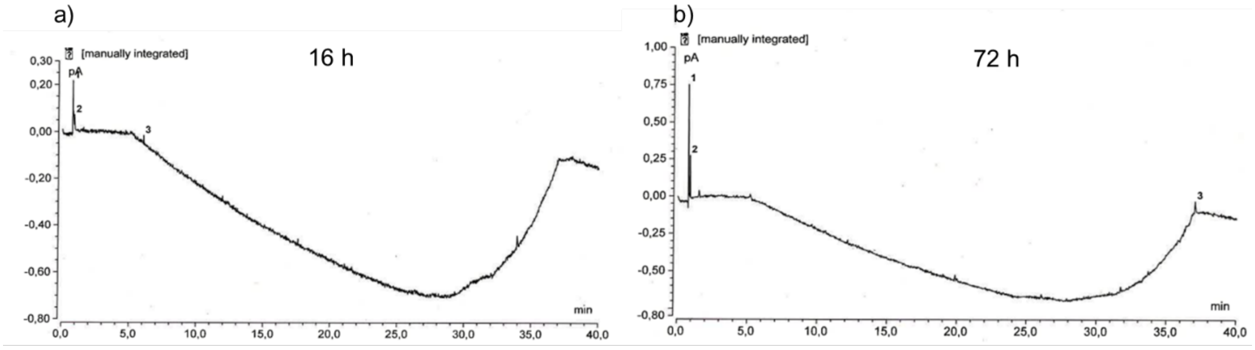

a)

No.	Ret.Time min	area-% %	Peak Name
1	0,78	79,34	Methan
2	0,90	17,77	Ethan
3	6,04	2,89	

b)

No.	Ret.Time min	area-% %	Peak Name
1	0,77	65,06	Methan
2	0,90	13,49	Ethan
3	37,04	21,46	

Instrument parameters:

Column:	27,5 m	HP-Plot/Al2O3 0,25/5,0df G/425	
Temperature:	220 / 40, 5 min iso 5/min 200, 3 min iso / 350		
Gas:	0,80 bar	H2	
Sample size:	500,0 µL	Split ratio:	20 : 1

Figure S18. Gas products of the reactions performed with Ni_3FeN/Ni_3Fe-350-2h under 25 bar of CO_2 at 100 ˚C for 16 h (a) and 72 h (b).

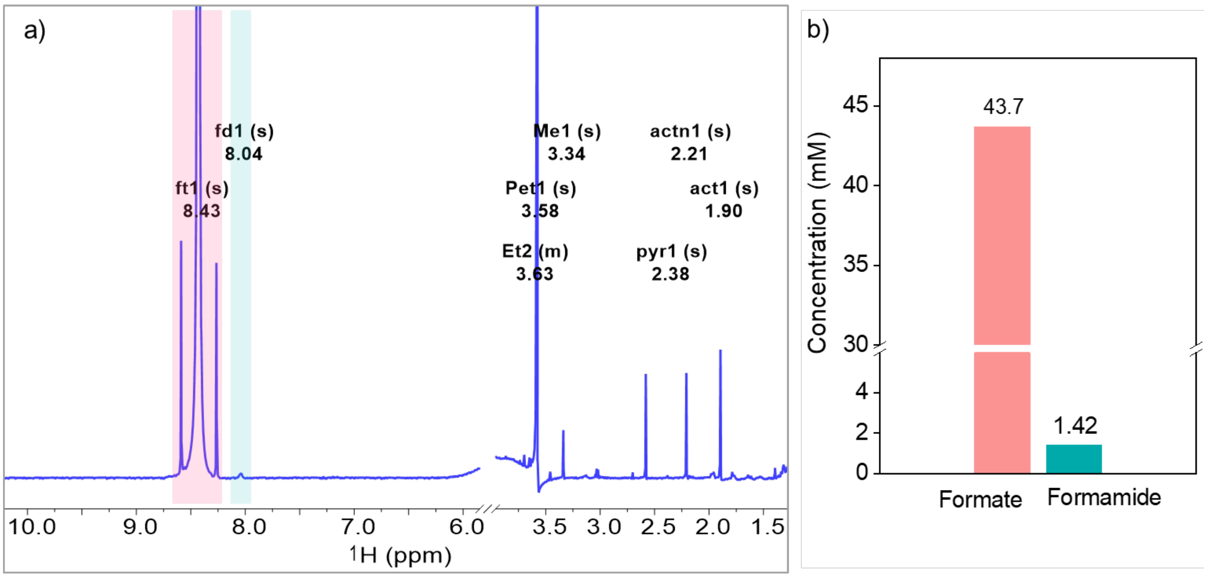

Figure S19. ^{1}H-NMR spectrum (a), and product concentrations (b) of the reaction performed with 25 bar CO_2 + H_2 mixture (3:2 ratio) at 100 ˚C for 16 h over Ni_3FeN/Ni_3Fe-350-2h heterostructure.

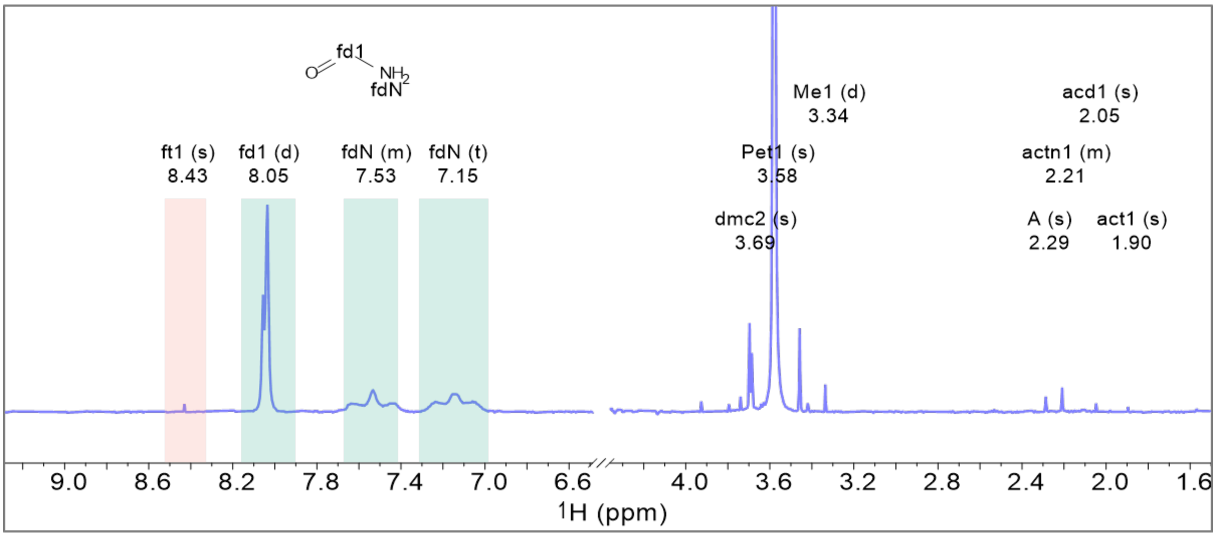

Figure S20. ^{1}H-NMR spectrum of formic acid (10 mM) conversion reaction at room temperature over Ni_3FeN/Ni_3Fe-350-2h particles for 16 h. ft: formate, fd: formamide, dmc: dimethylcarbonate, me: methanol, actn: acetone, acd: acetamide, act: acetate.

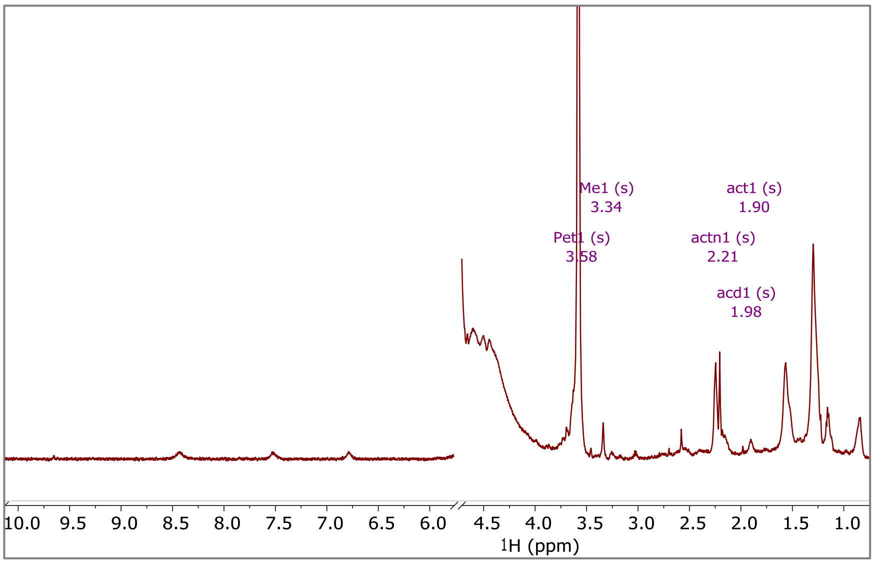

Figure S21. ^{1}H-NMR spectrum of the reaction performed with NH_4OH solution (1 mM) with Ni_3FeN/Ni_3Fe-350-2h nanoparticles under 25 bar of CO_2 at 100 ˚C for 16 h.

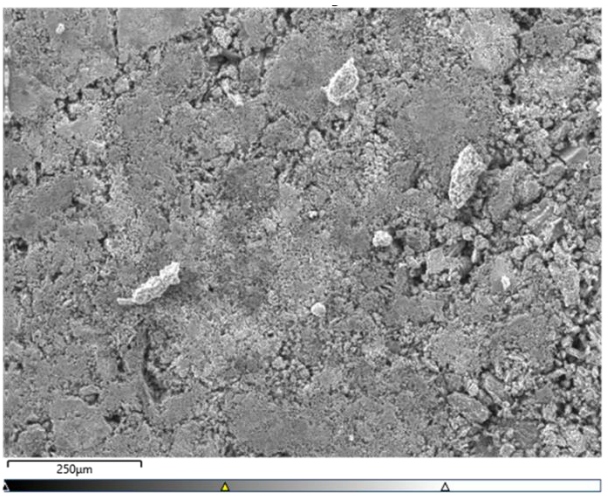

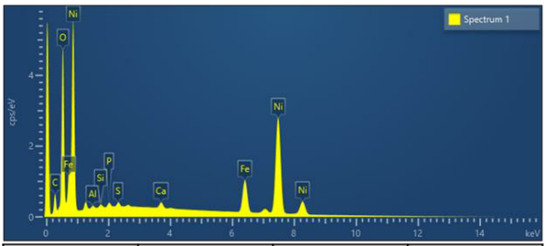

Element	Massen %	Massen % Sigma	Atom %
O	18.49	0.07	44.37
Fe	11.57	0.07	7.95
Ni	66.92	0.10	43.75
Mg	1.01	0.03	1.59
Al	0.24	0.02	0.34
Si	0.24	0.02	0.32
P	0.37	0.02	0.46
S	0.35	0.02	0.42
Cl	0.16	0.02	0.17
Ca	0.64	0.02	0.62

Figure S22. SEM-EDX results of Ni_3FeN/Ni_3Fe-350-2h sample after the reaction with 25 bar CO_2 at pH 6 and 100 ˚C for 16 h.

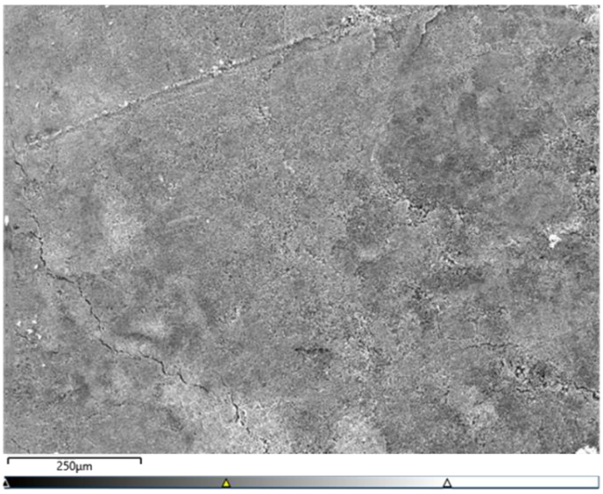

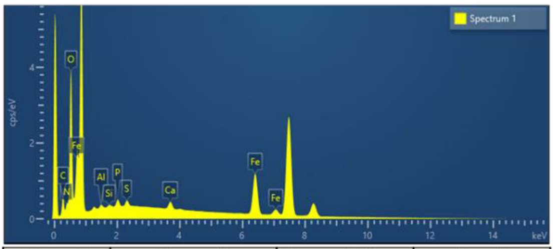

Element	Massen %	Massen % Sigma	Atom %
N	1.95	0.15	**5.35**
O	16.29	0.07	39.17
P	0.57	0.02	0.71
S	0.44	0.02	0.52
Ca	0.78	0.02	0.75
Fe	13.95	0.07	**9.61**
Ni	65.27	0.14	**42.77**
Mg	0.33	0.03	0.53
Al	0.29	0.02	0.42
Si	0.12	0.02	0.17

Figure S23. SEM-EDX results of Ni_3FeN/Ni_3Fe-350-2h sample after the reaction with 25 bar CO_2 at pH 6 and 25 ˚C for 16 h.

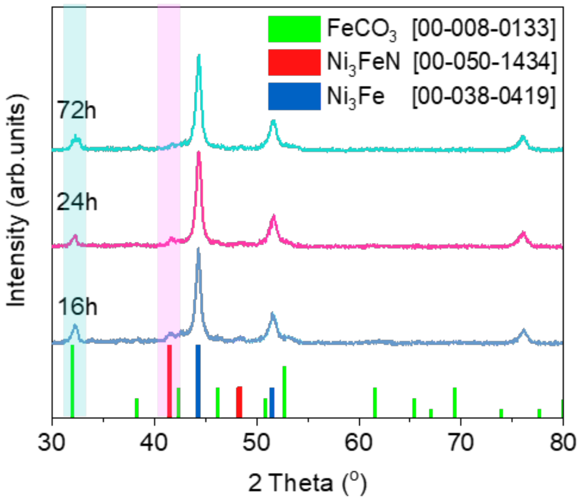

Figure S24. Post-reaction XRD patterns of Ni_3FeN/Ni_3Fe-350-2h after 16 h, 24 h, and 72 h with 25 bar of CO_2 at 100 ˚C.

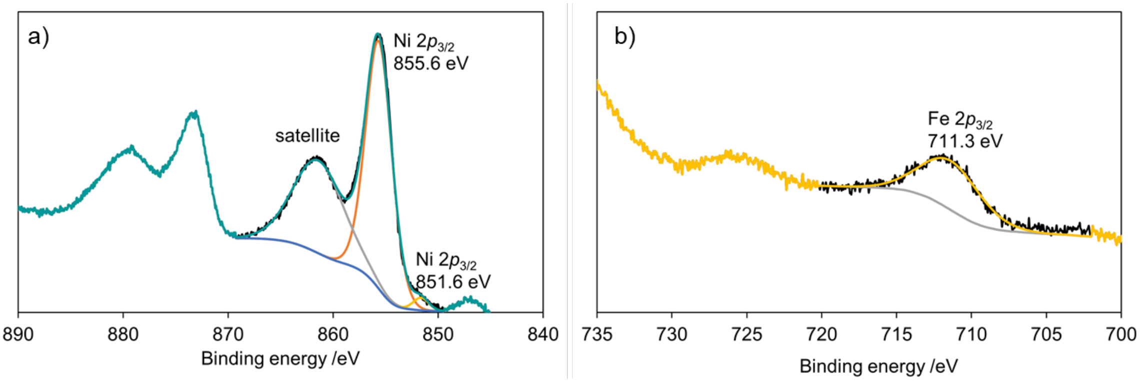

Figure S25. High-resolution XPS spectra of Ni 2*p* and Fe 2*p* of Ni_3FeN/Ni_3Fe-350-2h heterostructure after the reaction under 25 bar of CO_2 at 100 ˚C for 16 h.

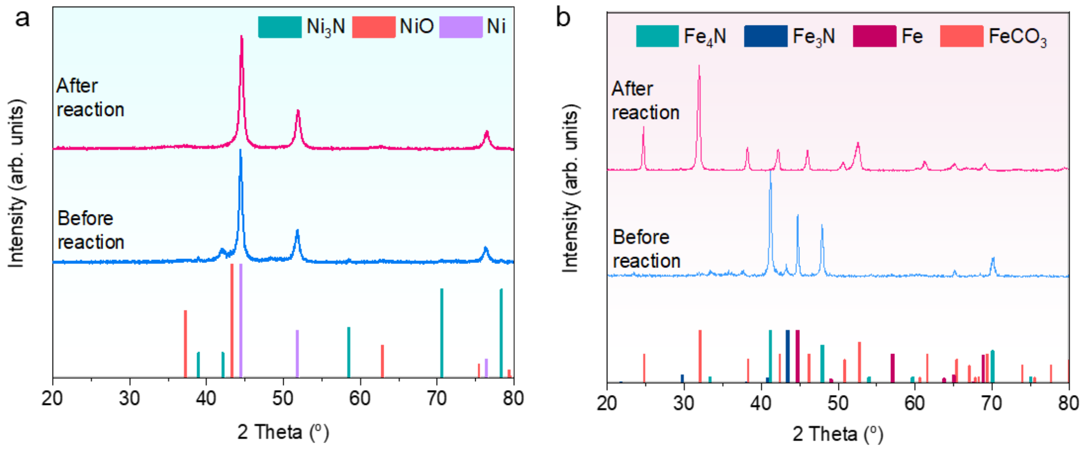

Figure S26. XRD patterns of Ni_3N/Ni (a) and Fe_xN (b) after the reaction under 25 bar of CO_2 at 100 ˚C for 16 h. Ni_3N PDF: 00-010-0280, Ni PDF: 03-065-2865, Fe_4N PDF: 00-006-0627, Fe_3N PDF: 00-049-1662, Fe PDF: 00-006-0696, $FeCO_3$ PDF: 00-038-0419.
